# ID helix-loop-helix proteins as determinants of cell survival in B-cell chronic lymphocytic leukemia cells *in vitro*

**DOI:** 10.1186/s12943-014-0286-9

**Published:** 2015-02-03

**Authors:** Sarah Weiler, Jolaolu A Ademokun, John D Norton

**Affiliations:** School of Biological Sciences, University of Essex, Colchester, Essex CO4 3SQ UK; Department of Haematology, Ipswich Hospital NHS Trust, Heath Road, Ipswich, Suffolk IP4 5PD UK

**Keywords:** Chronic lymphocytic leukemia, ID helix-loop-helix proteins, Cell survival/cell death, Drug resistance

## Abstract

**Background:**

Members of the inhibitor of DNA-binding (ID) family of helix-loop-helix proteins have been causally implicated in the pathogenesis of several types of B-cell lineage malignancy, either on the basis of mutation or by altered expression. B-cell chronic lymphocytic leukemia encompasses a heterogeneous group of disorders and is the commonest leukaemia type in the Western world. In this study, we have investigated the pathobiological functions of the ID2 and ID3 proteins in this disease with an emphasis on their role in regulating leukemic cell death/survival.

**Methods:**

Bioinformatics analysis of microarray gene expression data was used to investigate expression of *ID2*/*ID3* in leukemic versus normal B cells, their association with clinical course of disease and molecular sub-type and to reconstruct a gene regulatory network using the ‘maximum information coefficient’ (MIC) for target gene inference. *In vitro* cultured primary leukemia cells, either in isolation or co-cultured with accessory vascular endothelial cells, were used to investigate ID2/ID3 protein expression by western blotting and to assess the cytotoxic response of different drugs (fludarabine, chlorambucil, ethacrynic acid) by 3-(4,5-dimethylthiazol-2-yl)-2,5-diphenyltetrazolium bromide assay. ID2/ID3 protein levels in primary leukemia cells and in MEC1 cells were manipulated by transduction with siRNA reagents.

**Results:**

Datamining showed that the expression profiles of *ID2* and *ID3* are associated with distinct pathobiological features of disease and implicated both genes in regulating cell death/survival by targeting multiple non-overlapping sets of apoptosis effecter genes. Consistent with microarray data, the overall pattern of ID2/ID3 protein expression in relation to cell death/survival responses of primary leukemia cells was suggestive of a pro-survival function for both ID proteins. This was confirmed by siRNA knock-down experiments in MEC1 cells and in primary leukemia cells, but with variability in the dependence of leukemic cells from different patients on ID protein expression for cell survival. Vascular endothelial cells rescued leukemia cells from spontaneous and cytotoxic drug-induced cell death at least in part, via an ID protein-coupled redox-dependent mechanism.

**Conclusions:**

Our study provides evidence for a pro-survival function of the ID2/ID3 proteins in chronic lymphocytic leukemia cells and also highlights these proteins as potential determinants of the pathobiology of this disorder.

**Electronic supplementary material:**

The online version of this article (doi:10.1186/s12943-014-0286-9) contains supplementary material, which is available to authorized users.

## Introduction

The ‘Inhibitor of DNA-binding’ (ID) family of helix-loop-helix proteins function as key regulators of lineage specification and cell fate determination in Metazoa [1-3]. In mammals, there are four ID family members (ID1-ID4) that function by heterodimerising with and antagonising the activities of several classes of transcription factor. The E-protein family of basic helix-loop-helix transcription factors (E2A/TCF3, E2-2/TCF4 and HEB/TCF12) are the best characterised ID protein targets [1-4]. In hematopoietic cells, individual ID proteins perform distinct, but overlapping functions in a lineage- and differentiation-stage-specific manner [4-7]. ID proteins have also been causally implicated in the pathogenesis of leukemias and lymphomas; as in many solid tumour types, ID-mediated tumourigenesis is coupled to various oncogene/tumour suppressor pathways in hematopoietic cells [6]. Compelling evidence from loss- and gain-of-function studies in transgenic mice and cell line models supports a role for ID proteins in hematopoietic malignancies. Individual ID proteins have been ascribed either an ‘oncogene’ or ‘tumour suppressor’ function in primary human hematopoietic malignancies on the basis of expression level, mutational pattern and functional properties. For example, ID1 is a common downstream target of oncogenic tyrosine kinases, exemplified by BCR-ABL in chronic myeloid leukaemia, driving cell proliferation, survival and invasiveness [6]. High ID1 expression is also associated with a poor-prognosis subgroup of acute myeloid leukaemia [8]. Deregulated expression of ID2 is a consistent feature of Hodgkin’s lymphoma and appears to function in concert with ABF-1 in sequestering E2A and probably also PAX5 to augment the B-cell-specific gene regulatory programme in Hodgkin’s-Reed/Sternberg cells [9,10]. In Burkitt lymphoma by contrast, the function of the ID3 protein is recurrently inactivated through the acquisition of missense mutations in the *ID3* gene, predominantly affecting the helix-loop-helix dimerisation domain [11-13]. The *ID4* gene similarly behaves as a tumour suppressor through epigenetic silencing in most cases of acute myeloid leukemia [14], while in a sub-group of B-cell precursor acute lymphoblastic leukemia, expression of the *ID4* gene is deregulated by the recurrent t(6;14)(p22;q32) chromosomal translocation [15,16].

B-cell chronic lymphocytic leukemia (CLL) is the most prevalent type of leukemia in the Western world and it manifests as a clonal expansion of CD5^+^, CD19^+^, CD23^+^ B cells [17,18]. In this leukemia type, the status of only the ID4 family member has been evaluated in detail. In the Eμ-TCL1 mouse model of CLL, loss of an *ID4* allele leads to more aggressive disease while hemizygous loss of *ID4* in nontransformed TCL-1-positive B cells enhances cell proliferation [19]. These findings, together with the observation that *ID4* mRNA and protein expression is universally silenced in primary human CLL [14], strongly implicate ID4 as a tumour suppressor in this disease [19]. For the ID3 family member, microarray gene expression profiling data has shown that the expression of this gene is deregulated in CLL. An analysis of published microarray datasets of Zheng and colleagues [20] reveals a four-fold upregulation of *ID3* gene expression in CLL compared to normal CD5^+^ B-cells. An independent study [21] showed that *ID3* is among the most significantly overexpressed genes in a multivariate gene expression analysis comparing CLL with normal CD19^+^ B-cells, consistent with a potential role in CLL pathogenesis.

In addition to the various roles ascribed to individual ID proteins in regulating cell cycle/cell growth, differentiation, invasiveness, angiogenesis and metastasis in tumours of diverse histological origin, these proteins have also been widely documented to play a key role in regulating cell survival [1-4]. However, the behavior of individual ID proteins in functioning as either positive or negative regulators of cell viability is highly cell type-dependent, as illustrated by their contrasting functions in mediating cell survival or cell death in different solid tumour types in response to cytotoxic drugs [22-24] (and references therein). Since the primary phenotypic ‘defect’ in CLL cells is their impaired ability to undergo programmed cell death, and this has major implications for cytotoxic drug therapy [17,18], it was pertinent to determine whether ID proteins perform a functional role in regulating cell survival in this leukemia, particularly in response to cytotoxic drug treatment. We report here that the ID2 and ID3 proteins impart pro-survival functions in CLL cells cultured *in vitro*. In a more physiologically-relevant *in vitro* co-culture system, vascular endothelial cells rescue CLL cells from spontaneous and drug-induced cell death via an ID protein-coupled redox-dependent mechanism.

## Results

### Datamining of *ID2* and *ID3* microarray gene expression data in CLL

**Figure 1:**
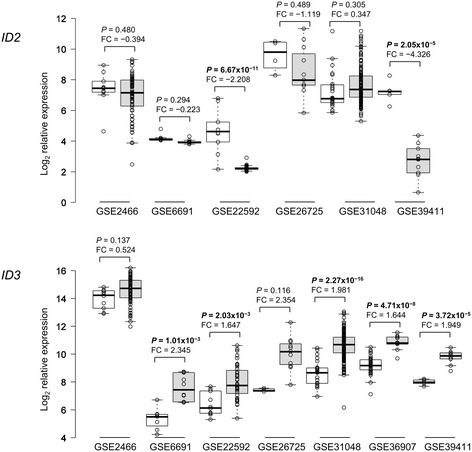
**Meta-analysis of microarray data comparing** ***ID2*** **/** ***ID3*** **gene expression in CLL with normal B cells.** For each dataset (identified by NCBI Gene Expression Omnibus GSE number), boxplot profiles are shown comparing normal B cells (open boxes) with CLL cells (shaded boxes). *P* values for the significance of difference in mean expression of the pair-wise comparisons were corrected for false discovery rate [51]. *P* values <0.05 are highlighted in bold-face. Log_2_ fold-change values (FC) are also given for each pair-wise comparison.

We initially extended previous findings from microarray data that reported up-regulation of *ID3* gene expression in CLL [20,21] by performing a systematic meta-analysis of microarray gene expression data, comparing relative levels of *ID2* and *ID3* in CLL versus normal B cells. In this and subsequent stages of our study, we confined the analysis to these two ID genes/proteins since previous studies have shown that *ID4* expression is very low in primary CLL [14,19] and in preliminary analysis we found that ID1 protein expression is undetectable by western blotting in CLL cells. As shown in Figure 1, *ID2* expression was down-regulated in CLL compared with normal B cells in five of six datasets analysed, with two of the five datasets showing a highly statistically significant difference in differential expression. By contrast, *ID3* expression was found to be up-regulated in CLL compared with normal B cells in all seven datasets analysed (Figure 1), consistent with previous studies [20,21]; in five datasets, the level of *ID3* up-regulation was statistically significant. Notably, the expression level of both *ID2* and *ID3* in CLL cells was extremely heterogeneous, extending over a wide range in each dataset.

**Figure 2:**
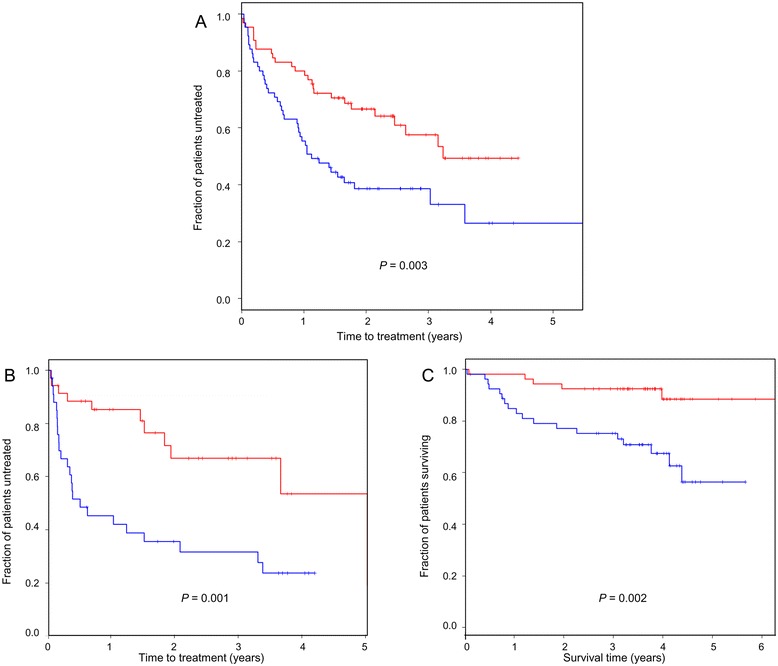
**Kaplan-Meier plots showing the relation between** ***ID2*** **expression and clinical outcome in CLL. A**: analysis of time to first treatment for GSE39671 dataset; **B**: analysis of time to first treatment for GSE22762 dataset; **C**: analysis of survival time for GSE22762 dataset. For each dataset, patients were grouped according to high (red line) and low (blue line) *ID2* expression. The significance of the difference in clinical end-point between high and low *ID2* expression patient groups was determined by log rank test.

We next analysed the relation between *ID* gene expression and clinical outcome in CLL using two independent cohort datasets for which patient annotation data is available in the public domain. As shown in Figure 2, high *ID2* expression was significantly associated with a more favourable disease course in terms of an overall longer time interval between initial sampling to first treatment for both cohorts. This pattern was reflected by the longer overall survival time for *ID2*-high patients (Figure 2C). No significant relation between *ID3* expression and these clinical end-points was observed with either dataset (Additional file 1: Figure S1).

**Figure 3:**
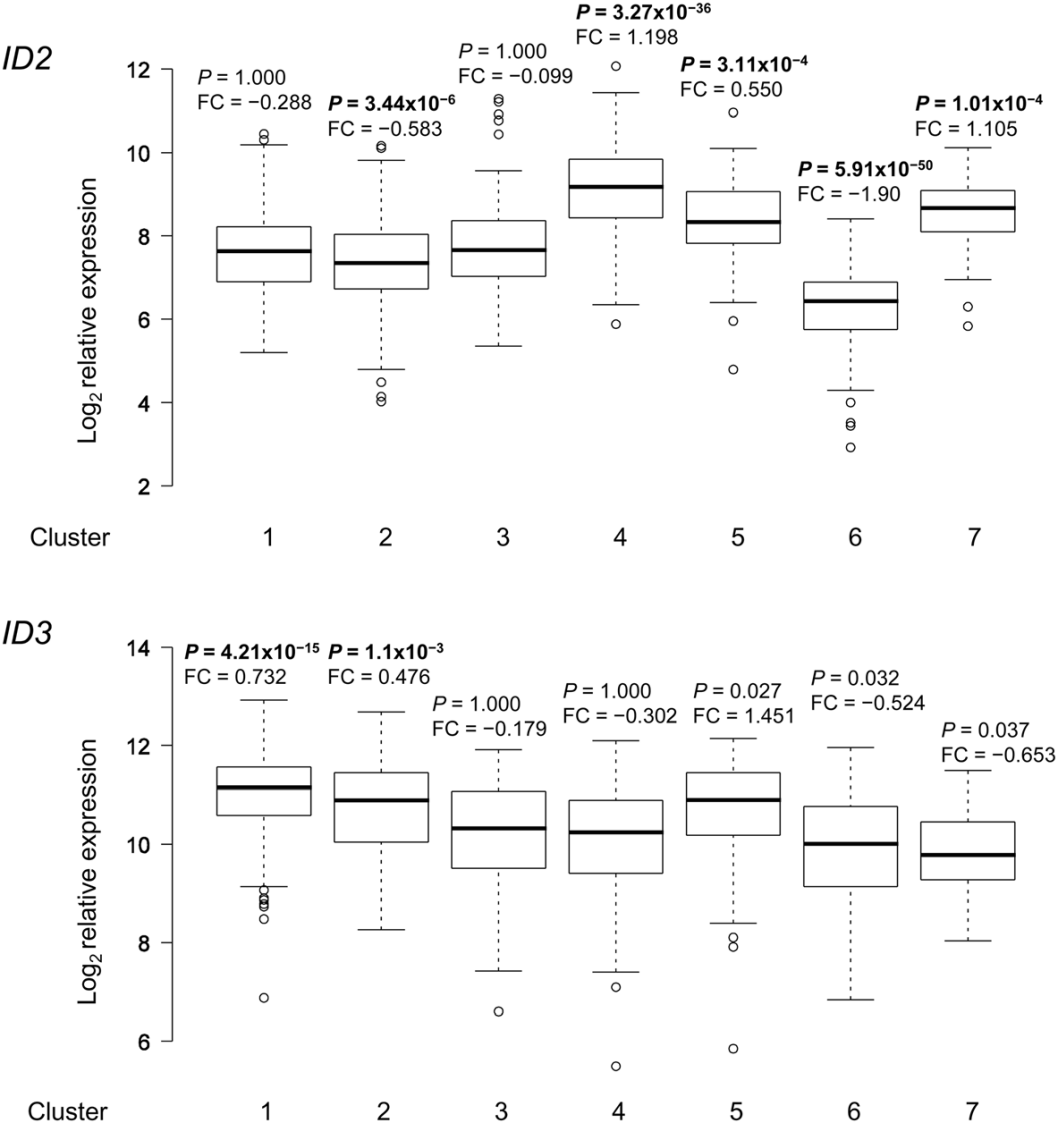
**Boxplot profile of** ***ID2*** **and** ***ID3*** **expression in the seven molecular sub-types of CLL identified by consensus clustering.** For each molecular sub-type (clusters 1–7), the distribution of relative *ID2*/*ID3* expression values is shown together with the log_2_ fold-change (FC) and significance level of the difference between the mean expression for the sub-type compared to all other sub-types. Significant differences that indicate over- or under-expression of Id genes in individual sub-types are highlighted in bold-face. *P* values were corrected for false discovery rate [51].

Recently, Friedman et al. [25] defined seven distinct molecular sub-types of CLL on the basis of unsupervised clustering of gene expression microarray data compiled from 15 datasets representing 893 unique CLLs. To determine whether differences in *ID2*/*ID3* expression pattern are associated with distinct molecular sub-types in CLL, we performed a similar unsupervised consensus clustering analysis of 871 CLLs compiled from 14 microarray datasets available in the public domain (see Materials and methods). As shown in Additional file 2: Figure S2, consensus clustering defined seven as the optimum number of cluster groups in this combined, 871 sample dataset. The gene signatures defining each of these cluster groups mapped onto an overlapping set of pathways and oncogenic signatures (Additional file 3: Table S1; Additional file 4: Table S2) similar to those reported previously [25]. Figure 3 shows the gene expression profiles of *ID2* and *ID3* across the seven cluster groups. There were significant differences in *ID2*/*ID3* expression levels amongst the different cluster groups. *ID2* was most significantly up-regulated in cluster group 4 while *ID3* was most significantly up-regulated in cluster group 1 (Figure 3 and see Additional file 3: Table S1; Additional file 4: Table S2).

**Figure 4:**
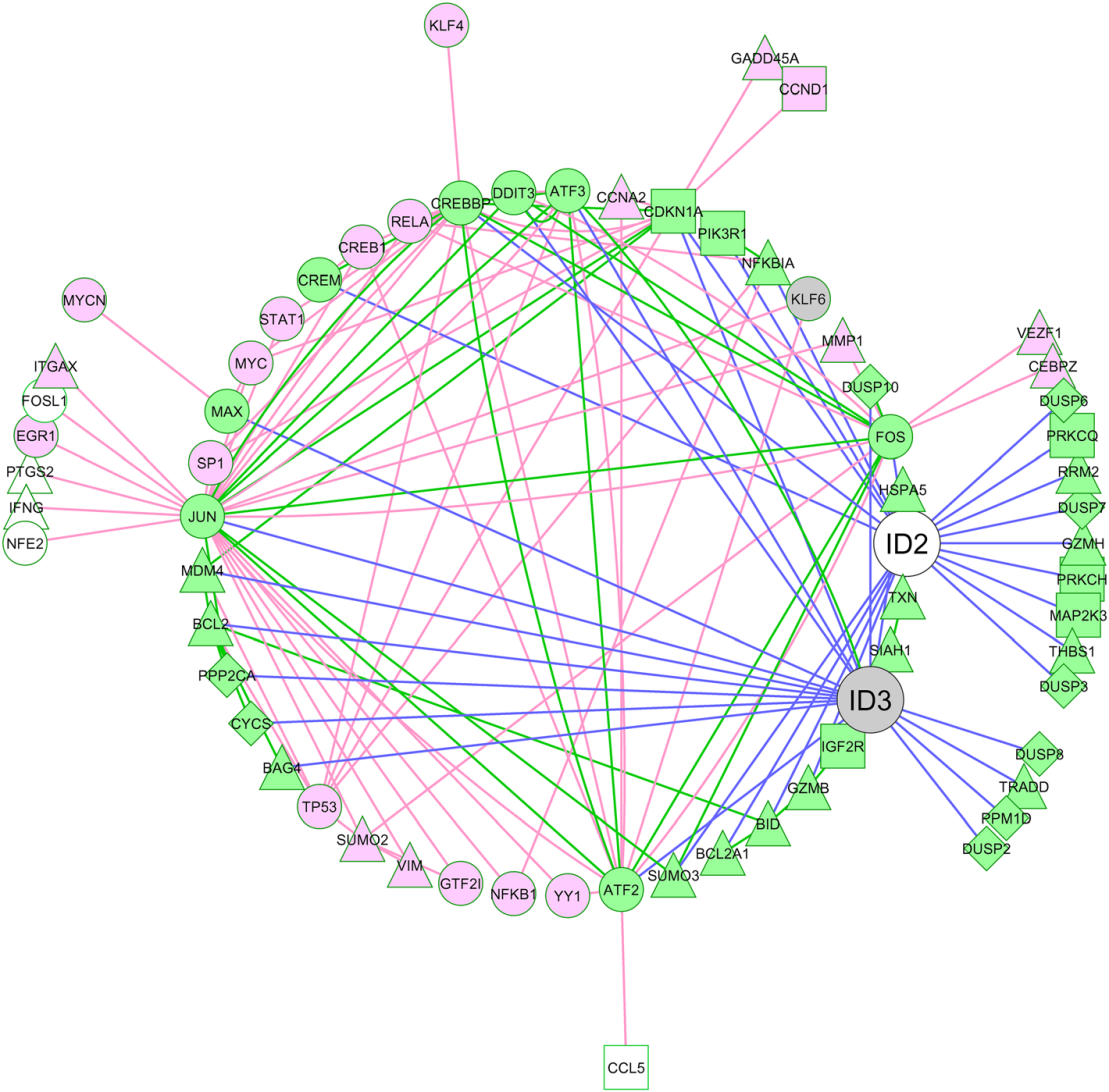
**Network graph showing interactions between ID2 and ID3 and their MIC-inferred ‘target’ genes regulating cell death.** MIC-inferred apoptosis target genes, denoted by green nodes connected to ID2/ID3 by blue edges, were compiled from apoptosis pathway target genes in Additional file 6: Table S4. Green edges define protein-protein interactions, curated from the String database [28]. Pink edges denote literature-validated regulatory interactions mined from the UniHI database [29] that have been expanded to include first-neighbour interactions with additional targets as either pink nodes (not MIC-inferred target genes) or as grey (ID3) or white (ID2) MIC-inferred target genes listed in Additional file 5: Table S3. A Cytoscape network graph circle layout is shown.

To gain insight into the biological functions of the ID2 and ID3 proteins in CLL, we employed an unsupervised reverse engineering of gene regulatory networks approach by determining pair-wise statistical dependences between expression of *ID2*/*ID3* and all other genes in the combined, 871 sample dataset (above). For this, we used the Maximum Information Coefficient (MIC) algorithm [26] which we and others have previously shown to out-perform more commonly used metrics such as linear regression and mutual information [26,27]. MIC analysis identified 678 (*ID2*) and 517 (*ID3*) regulatory interactions with unique genes (Additional file 5: Table S3) with only 21 genes in common. This mutual exclusivity of MIC-inferred regulatory interactions of the two ID genes was highly statistically significant (*P* <0.001 by hypergeometric distribution test). Despite this lack of overlap, the lists of MIC-inferred ‘target’ genes of the two ID proteins were both over-represented in many of the same Gene Ontology biological processes and pathways (Additional file 6: Table S4), in accord with the known functional properties of these ID proteins [1-3]. Several of these pathways (eg apoptosis, TGF-alpha signalling, oxidative stress, angiogenesis, VEGF signalling, p53 pathway) are known to play a key role in the pathogenesis of CLL [18] (and references therein). We focussed on apoptosis as the top-ranked pathway and second-ranked Gene Ontology term for both *ID* genes (Additional file 6: Table S4). Two additional apoptosis pathways, oxidative stress response and the p53 pathway were also shared by the MIC-inferred gene lists of ID2 and ID3. Figure 4 shows a network graph view of the inferred regulatory interactions between ID2/ID3 and their apoptosis ‘target’ genes, compiled from the gene lists that overlap with the three apoptosis pathways (Additional file 6: Table S4). The MIC-inferred apoptosis genes comprise both positive and negative regulators of cell death. The network in Figure 4 has been expanded to include documented protein-protein [28] and literature-validated regulatory interactions [29] and reveals a high degree of inter-connectivity between the mutually exclusive MIC-inferred ID2 and ID3 putative ‘target’ genes. Collectively, these data imply a key role for both ID2 and ID3 in regulating cell death/survival in CLL, most likely be targeting multiple independent sets of target genes that are shared by the same pathways.

### Expression of ID proteins in primary CLL and correlation with *in vitro* drug sensitivity

**Figure 5:**
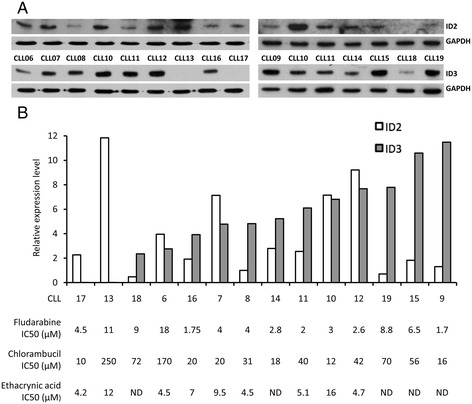
**Analysis of ID protein expression levels in primary CLL. (A)** Western blotting analysis of ID2 and ID3 expression was performed in two separate experiments depicted in the two panels shown. CLL10 and CLL11 samples were included in both experiments to monitor internal consistency. Immunoblots were re-probed with anti-GAPDH antibody as a control for protein loading. The original western blot images that were used to compile Figure 5A are shown in Additional file 8: Figure S3. **(B)** Protein bands were quantified by densitometric scanning and normalized to the GAPDH loading control and, for each ID protein, expressed as fold-change (relative expression level) relative to the CLL sample with the lowest expression level on the left blot (CLL08 for ID2 and CLL17 for ID3). Densitometric quantification of the western blotting data is shown in Additional file 9: Figure S4. CLL samples are shown rank-ordered by increasing levels of ID3 expression. *In vitro* IC_50_ values were determined following 72 hrs of treatment for each CLL sample; ND: not determined.

**Figure 6:**
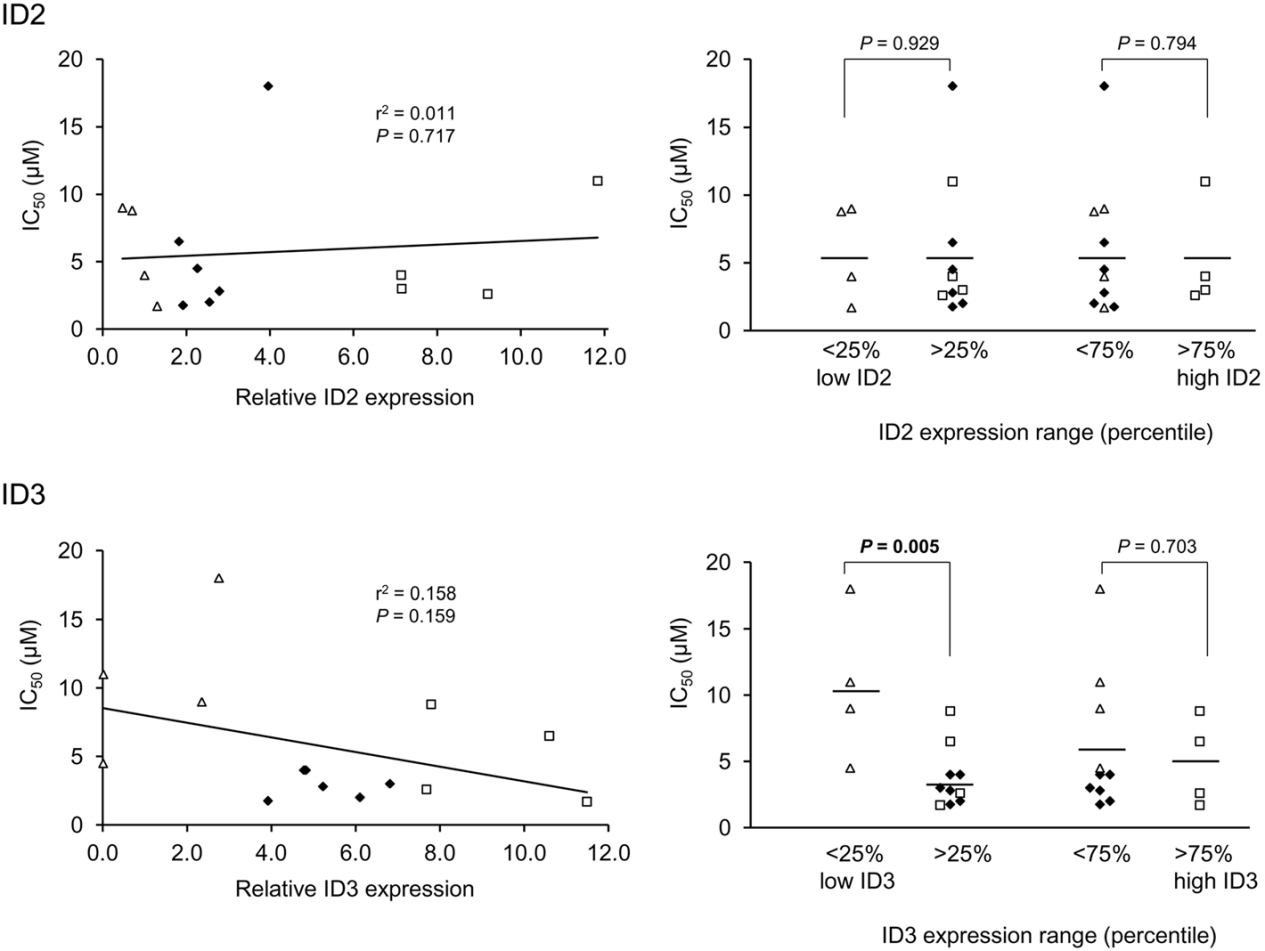
**Relation between** ***in vitro*** **fludarabine resistance and ID protein expression levels in 14 CLL samples.** Data for relative ID expression levels and IC_50_ values are given in Figure 5B in the main manuscript. The left-hand plots show linear regression analysis where samples are coded according to ID protein expression range (open triangles: lowest 25th percentile; open squares: highest 75th percentile; solid diamonds: remaining samples). The right-hand plots show a comparison of ID protein expression levels between samples grouped according to percentile range of ID protein expression. Significant (<0.05) *P* values, calculated by Student’s *t*-test, are indicated in bold-face.

**Figure 7:**
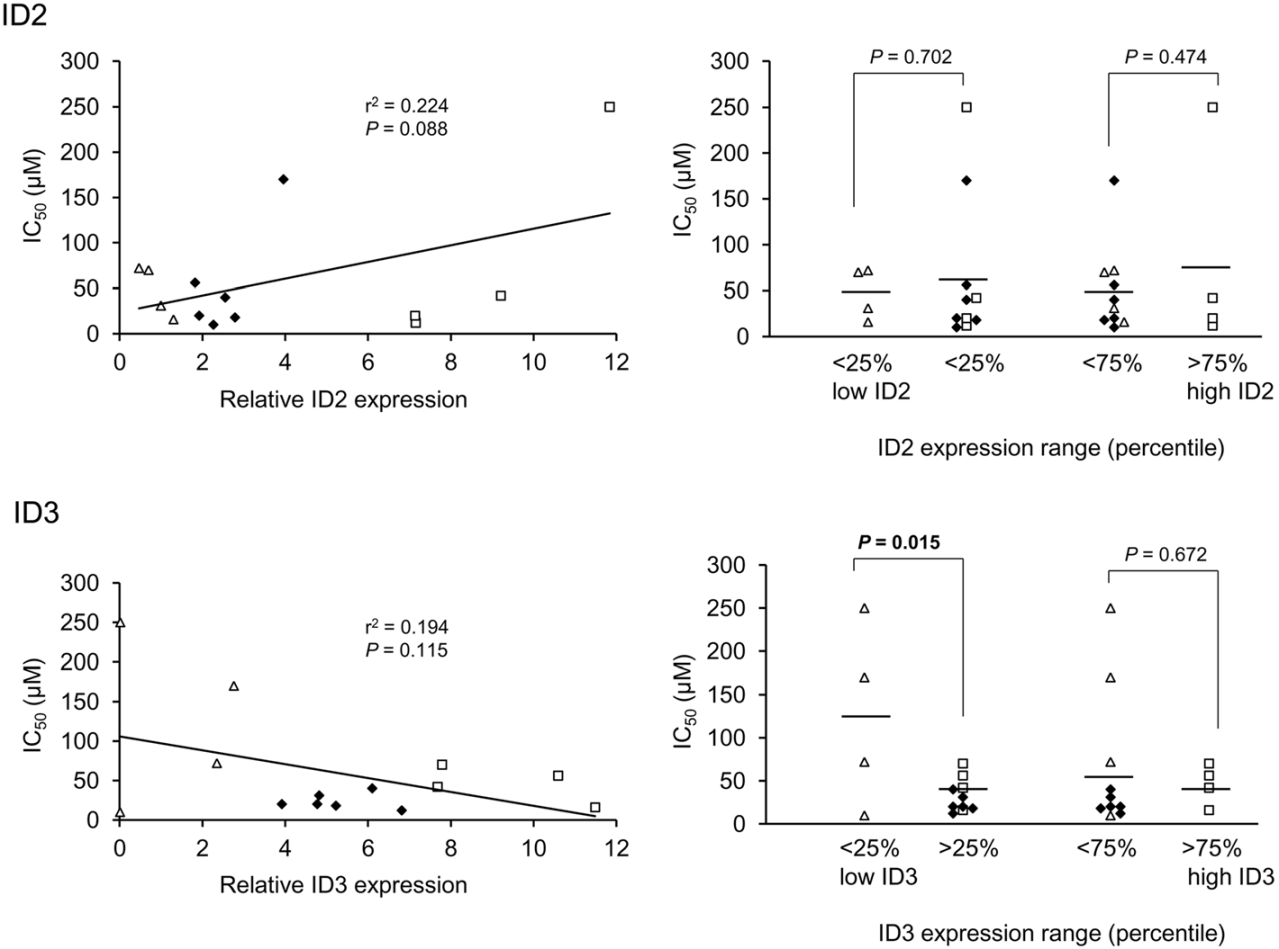
**Relation between** ***in vitro*** **chlorambucil resistance and ID protein expression levels in 14 CLL samples.** Data for relative ID expression levels and IC_50_ values are given in Figure 5B in the main manuscript. The left-hand plots show linear regression analysis where samples are coded according to ID protein expression range (open triangles: lowest 25th percentile; open squares: highest 75th percentile; solid diamonds: remaining samples). The right-hand plots show a comparison of ID protein expression levels between samples grouped according to percentile range of ID protein expression. Significant (<0.05) *P* values, calculated by Student’s *t*-test, are indicated in bold-face.

**Figure 8:**
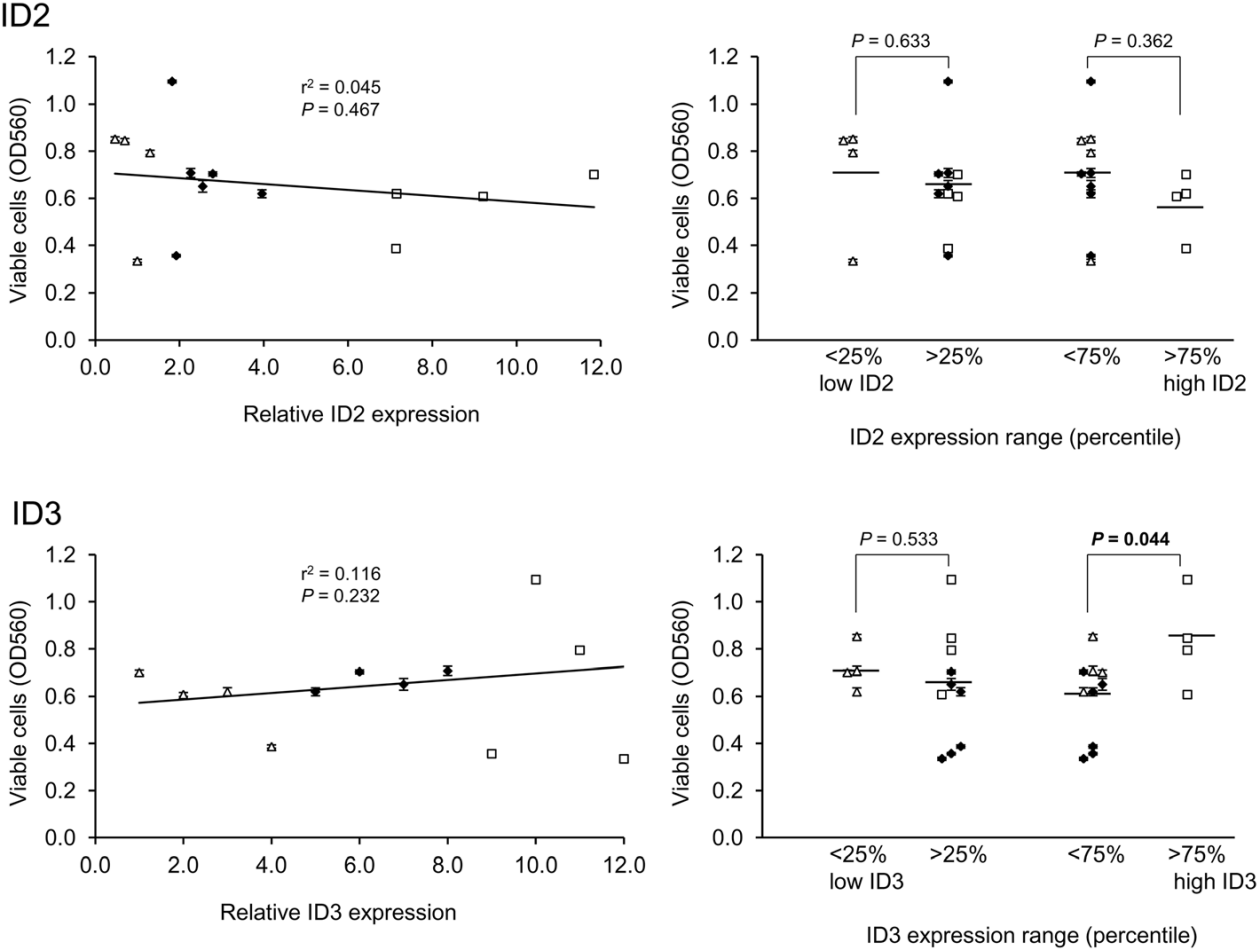
**Relation between resistance to** ***in vitro*** **spontaneous cell death and ID protein expression levels in 14 CLL samples.** Relative numbers of viable cells were determined from MTT assay data after 72 hrs culture; data points show the mean ± SEM. Data for relative ID expression levels are given in Figure 5B in the main manuscript. The left-hand plots show linear regression analysis where samples are coded according to ID protein expression range (open triangles: lowest 25th percentile; open squares: highest 75th percentile; solid diamonds: remaining samples). The right-hand plots show a comparison of ID protein expression levels between samples grouped according to percentile range of ID protein expression. Significant (<0.05) *P* values, calculated by Student’s *t*-test, are indicated in bold-face.

To validate and extend the preceding bioinformatics data, we initially analysed the expression of the ID2 and ID3 proteins in a series of 14 CLL (Additional file 7: Table S5) for which *in vitro* toxicity data was obtained for three different drugs: fludarabine, chlorambucil and ethacrynic acid. The first two of these drugs are widely used as part of standard chemotherapy for CLL whilst ethacrynic acid displays potent and specific *in vitro* cytotoxicity against CLL cells through inhibition of the Wnt/β-catenin signalling pathway [30]. Wnt/β-catenin signalling is reportedly a key regulator of *ID* gene expression [2,3] and at least for *ID2*, components of this pathway were significantly over-represented in the list of MIC-inferred regulatory interactions for *ID2* (see Additional file 6: Table S4). The results of western blotting analysis of the 14 CLL are shown in Figure 5A (and see Additional file 8: Figure S3), and, in Figure 5B, the normalised expression levels of ID2 and ID3 are displayed graphically together with IC_50_ values for each of the three drugs. Densitometric quantification of the western blotting data is shown in Additional file 9: Figure S4. The most obvious observation from these data is the extreme heterogeneity in ID protein expression pattern amongst different CLL samples, consistent with the microarray data (see Figure 1). There was no significant correlation between the expression levels of the two ID proteins (*P* = 0.39; Pearson’s correlation). However, as shown in Figure 6 for fludarabine and in Figure 7 for chlorambucil, while there was no significant correlation between ID2/ID3 expression and drug IC_50_ values by Pearson’s analysis, those CLLs expressing the lowest 25th percentile range of ID3 (but not of ID2) protein were significantly more resistant than other CLLs to both drugs. By contrast, a similar analysis of cell death, in the absence of drug treatment showed that those CLLs expressing the highest 75th percentile range of ID3 (but again, not of ID2) were significantly more resistant than other CLLs to ‘spontaneous’ cell death (Figure 8).

### Changes in ID2 and ID3 protein expression pattern following *in vitro* drug treatment

**Figure 9:**
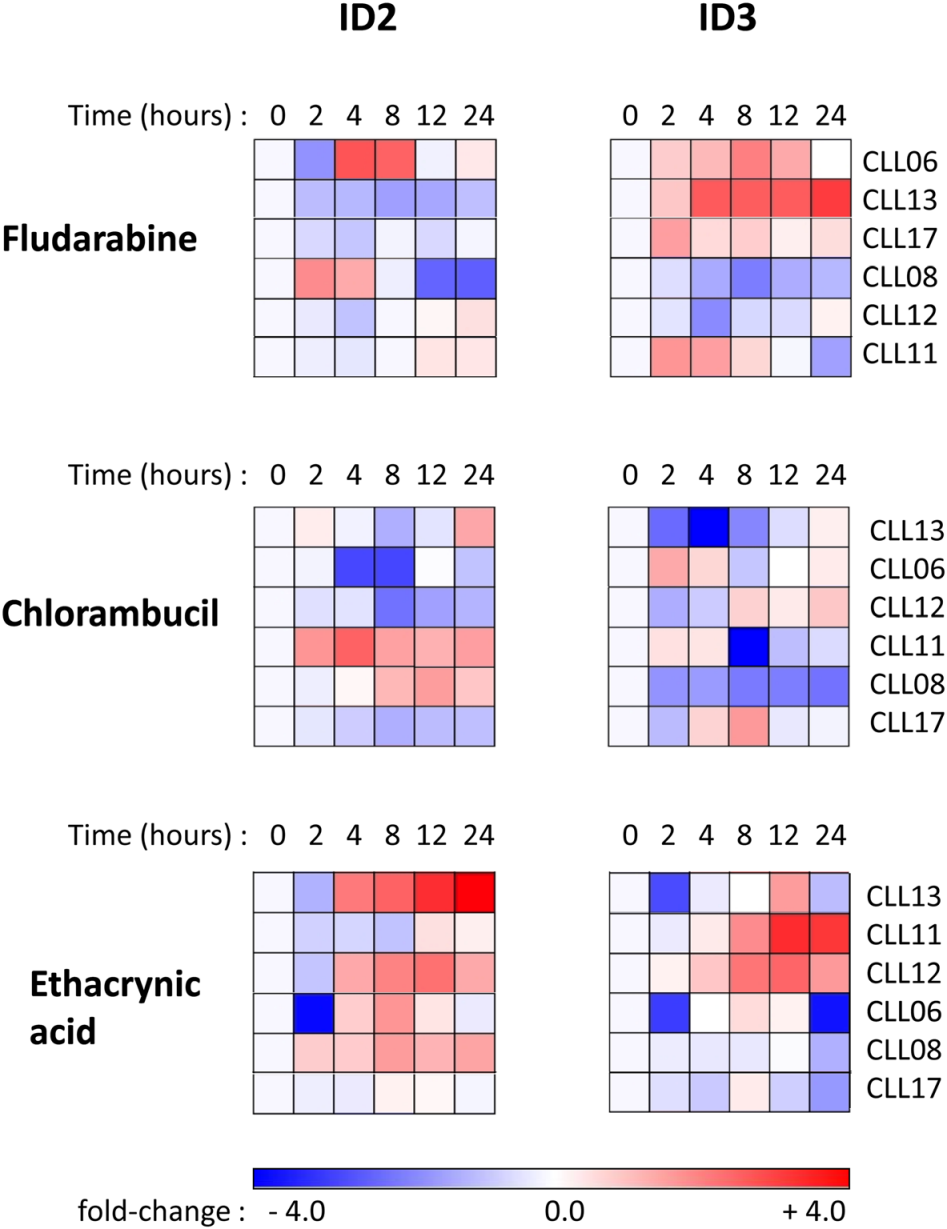
**ID2 and ID3 protein expression patterns in response to** ***in vitro*** **drug treatment.** CLL cells from six patients were cultured in the presence of varying concentrations of fludarabine, chlorambucil and ethacrynic acid for 24 hrs. The concentration of drugs were chosen for each CLL, based on IC_50_ values (see Figure 5B) such that a significant fraction of viable cells remained after the 24 hrs drug treatment period. At the indicated time points, ID2, ID3 and GAPDH (control) levels were analysed by western blotting. The bands were quantified by densitometric scanning and normalized to the intensity of the loading control (GAPDH). The fold-change in expression, relative to the untreated control was determined and, after log2-transformation was displayed as a heat map as shown. For each drug, CLL samples are shown rank-ordered by decreasing IC_50_ value (decreasing drug resistance).

In understanding how levels of ID2/ID3 protein impact on the *in vitro* drug response, it was important to determine whether the expression pattern of these proteins undergoes any change following drug treatment. As shown by the heat-map representation of time-course western blot experiments in Figure 9, both ID proteins displayed dynamic changes in expression, with each drug eliciting a distinct pattern of ID protein expression. The expression dynamics of ID2/ID3 were extremely heterogeneous with no two CLLs displaying an identical pattern. However, closer inspection reveals some suggestive trends in the ID protein expression dynamics in the more drug-resistant CLLs in response to all three drugs. In Figure 9, the CLLs are arranged in ranked order of decreasing IC_50_ value (decreasing drug resistance) for each drug.

In five of the six CLLs examined, there was either a net down-regulation or else a very modest change in expression of ID2 following 24 hrs of fludarabine treatment. CLL06, which was the most resistant to this drug (IC_50_ = 18 μM - see Figure 5B), was exceptional in displaying a transient peak of increased expression at 4-8 hrs. CLL06, together with the second most fludarabine-resistant sample in the series, CLL13, (IC_50_ = 11 μM) also displayed the highest sustained increase in expression of ID3. The third most fludarabine-resistant sample (CLL17; IC_50_ = 4.5 μM) similarly exhibited a sustained, although less pronounced increase in ID3 expression. In the remaining (more fludarabine-sensitive) CLLs, there was either a net down-regulation or else very little change in ID2/ID3 expression beyond 4 hrs of drug treatment. Also, those CLLs that initially expressed low levels of ID3 (including the two fludarabine-resistant samples, CLL06 and CLL13 – see Figure 5B) were characterised by a sustained pattern of increased ID3 expression in response to fludarabine while those CLLs expressing high levels of ID3 before drug treatment exhibited the most down-regulation of ID3 expression. Thus, the low ID3 expression associated with fludarabine-resistance before drug treatment (Figure 6), manifested as increased expression of ID3 on exposure to this drug. A similar pattern was observed for ID3 in relation to chlorambucil response (Figure 9); the three more resistant CLLs (CLL13, CLL06, CLL12) all displayed a sustained increase of ID3. However, two of the more chlorambucil-sensitive CLLs (CLL11, CLL08), in addition to CLL13, showed sustained up-regulation of ID2 in response to chlorambucil, although we note that both CLL11 and CLL08 exhibited the most pronounced down-regulation of ID3 in response to this drug (Figure 9).

Ethacrynic acid treatment resulted in up-regulation of ID2/ID3 in four of the six CLL samples (Figure 9). However, as with fludarabine, the most marked up-regulation was seen in those CLLs that were most resistant to this drug: CLL13 (ID2) and CLL11, CLL12 (ID3). The remaining more ethacrynic acid-sensitive CLLs (CLL06, CLL08, CLL17) all displayed a strong down-regulation in ID3 expression and only minor changes in expression of ID2.

Collectively, the above time-course ID protein expression data suggest a trend in which for each drug, the more drug-resistant CLLs were associated with a pattern of marked up-regulation of ID3 and to a lesser extent of ID2.

### siRNA-mediated knock-down of ID2 and ID3 expression reduces survival of CLL cells

**Figure 10:**
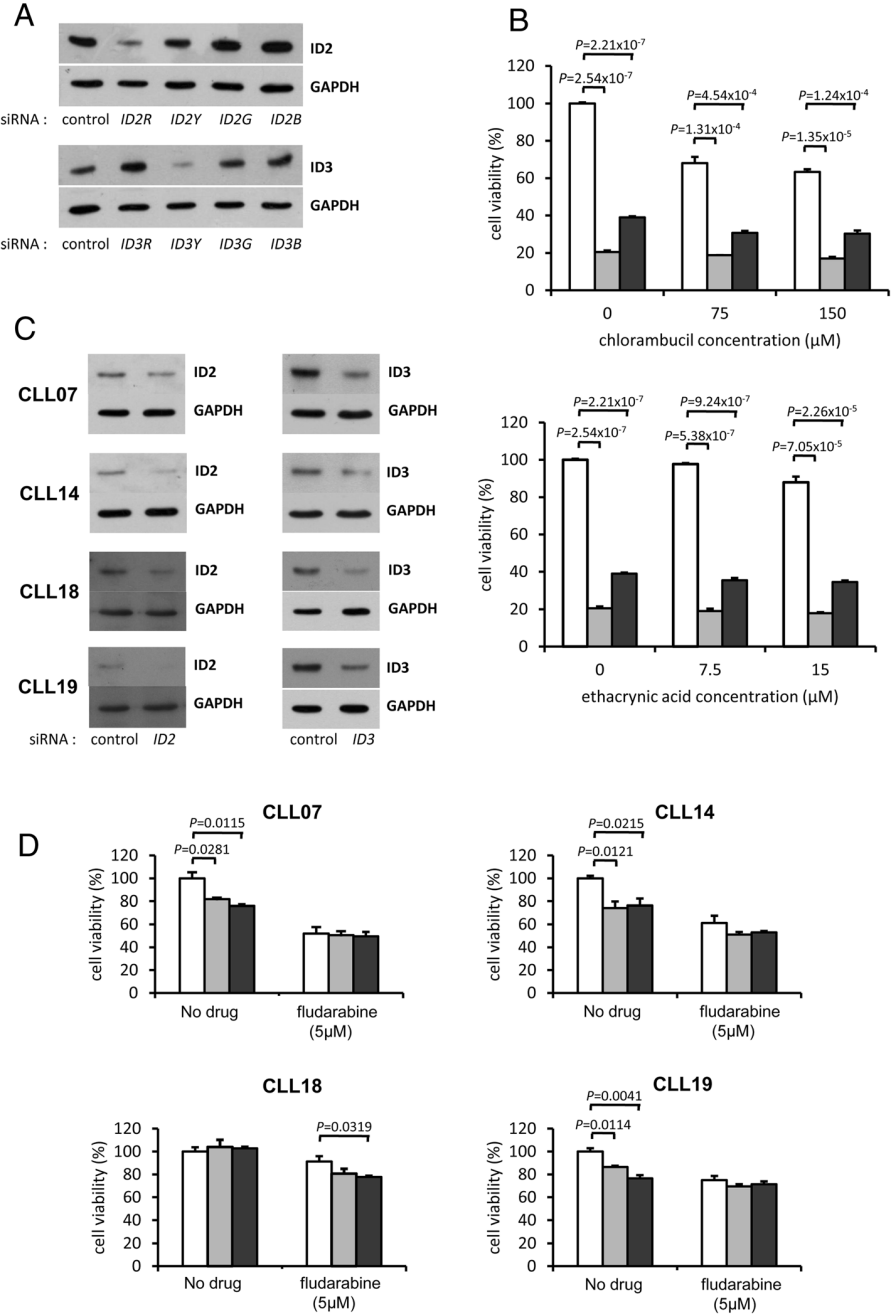
***ID2*** **and** ***ID3*** **gene knockdown reduces survival of MEC1 cell line and primary CLL cells. (A)** Western analysis of MEC1 cells infected with lentiviruses encoding either a control siRNA sequence or encoding one of four siRNAs targeting different sequences in the *ID2/ID3* mRNAs. The original western blot images are shown in Additional file 10: Figure S5. **(B)** MEC1 cells that were transduced with control siRNA (open bars), *ID2R*-siRNA (light-shaded bars) or *ID3Y*-siRNA (dark-shaded bars) were incubated in the absence or presence of increasing concentrations chlorambucil or ethacrynic acid for 48 hrs and cell viability was assessed by MTT assay. The mean ± SEM of three independent samples is shown. **(C)** CLL cells from four patients were transfected with 60 nM negative control siRNA or with *ID2/ID3* siRNA. 72 hrs post-transfection, cells were harvested and analysed by western blotting. The original western blot images are shown in Additional file 10: Figure S5. **(D)** 1×10^6^ cells from the four CLL patients were transfected with 60 nM negative control siRNA (open bars) or with the same concentration of *ID2* siRNA (light-shaded bars) or *ID3* siRNA (dark-shaded bars). 48 hrs post-transfection, the cells were incubated in the absence or presence of fludarabine (5 μM) for a further 24 hrs. Cell viability was assessed by MTT assay and values were normalised to the untreated control siRNA sample. Data from the mean ± SEM from three independent samples is shown.

The observed pattern of drug-induced expression changes of ID proteins in CLL, in which down-regulation was associated with cell death in more drug-sensitive CLLs and up-regulation with cell survival in the more drug-resistant CLLs is consistent with the data showing high ID3 expression in CLLs that were more resistant to spontaneous cell death (Figure 8) and is suggestive of a pro-survival function for the ID proteins in this tumour type. To directly evaluate this, we initially employed MEC1 cells [31] as a cell line model of CLL. As shown in Figure 10A (and Additional file 10: Figure S5), following transduction of these cells with four different lentivirus vectors targeting each *ID* mRNA, a single cell pool of MEC1 cells was identified for each *ID* gene that displayed reduced levels of expression of ID protein (ID2R and ID3Y). These cells were expanded alongside the control siRNA-transduced cells (Figure 10A) and examined for *in vitro* drug sensitivity. Since MEC1 cells are known to be recalcitrant to fludarabine-induced cell death [32], the impact of either ID2 or ID3 knock-down on chlorambucil and ethacrynic acid-induced cell death was evaluated. As shown in Figure 10B (and Additional file 10: Figure S5), even in the absence of drug, the viability of *ID2R*- *ID3Y*-transduced cells was significantly less than control cells. Thus, loss of either ID2 or ID3 expression leads to extensive cell death. Following chlorambucil/ethacrynic acid treatment, there was a commensurate further loss of viability of *ID2R*- *ID3Y*-transduced cells, compared with control cells (Figure 10B).

This analysis was extended to four different primary CLLs by using siRNA reagents to directly knock-down the expression of ID2 or ID3. As shown in Figure 10C (and in Additional file 10: Figure S5), treatment of CLL cells with the *ID2* or *ID3* siRNAs led to a variable reduction in levels of the respective proteins, relative to control siRNA. Since the preceeding analysis showed that fludarabine elicited the most distinctive pattern of association between drug-induced ID2/ID3 expression and fludarabine resistance (Figure 9), we focussed on this drug in analysing the effects of ID2/ID3 knock-down. Figure 10D shows that even in the absence of fludarabine, in three out of the four CLL samples examined, knockdown of ID2 and ID3 reduced cell survival significantly. In CLL18, knockdown of ID2 or ID3 had no significant effect on cell viability in the absence of fludarabine treatment (Figure 10D). Of the four CLLs investigated, this CLL was the most resistant to fludarabine (IC_50_ = 9.0 μM). Following treatment of CLL18 cells with fludarabine, cell viability was reduced by 8.8% in the control sample compared to 21% in the ID2 knock-down and to 24% in the ID3 knockdown sample. Although the magnitude of this siRNA effect only reached statistical significance in the latter (Figure 10D), these data suggest that in this more fludarabine-resistant CLL, in which the overall extent of fludarabine-induced cell death was less than in the three other CLLs, loss of ID protein function demonstrably potentiates the fludarabine cytotoxic response. siRNA-mediated knock-down of ID2 and ID3 led to a modest (not statistically significant) reduction in cell viability when compared to control siRNA-treated cells following fludarabine treatment in two of the other CLLs (CLL14, CLL19).

Taken together, these observations in primary CLL cells are consistent with the MEC1 cell line stable knock-down data in showing that the ID2 and ID3 proteins perform a pro-survival function in CLL.

### Vascular endothelial cells rescue CLL cells from spontaneous and drug-induced cell death via an ID protein-coupled redox-dependent mechanism

**Figure 11:**
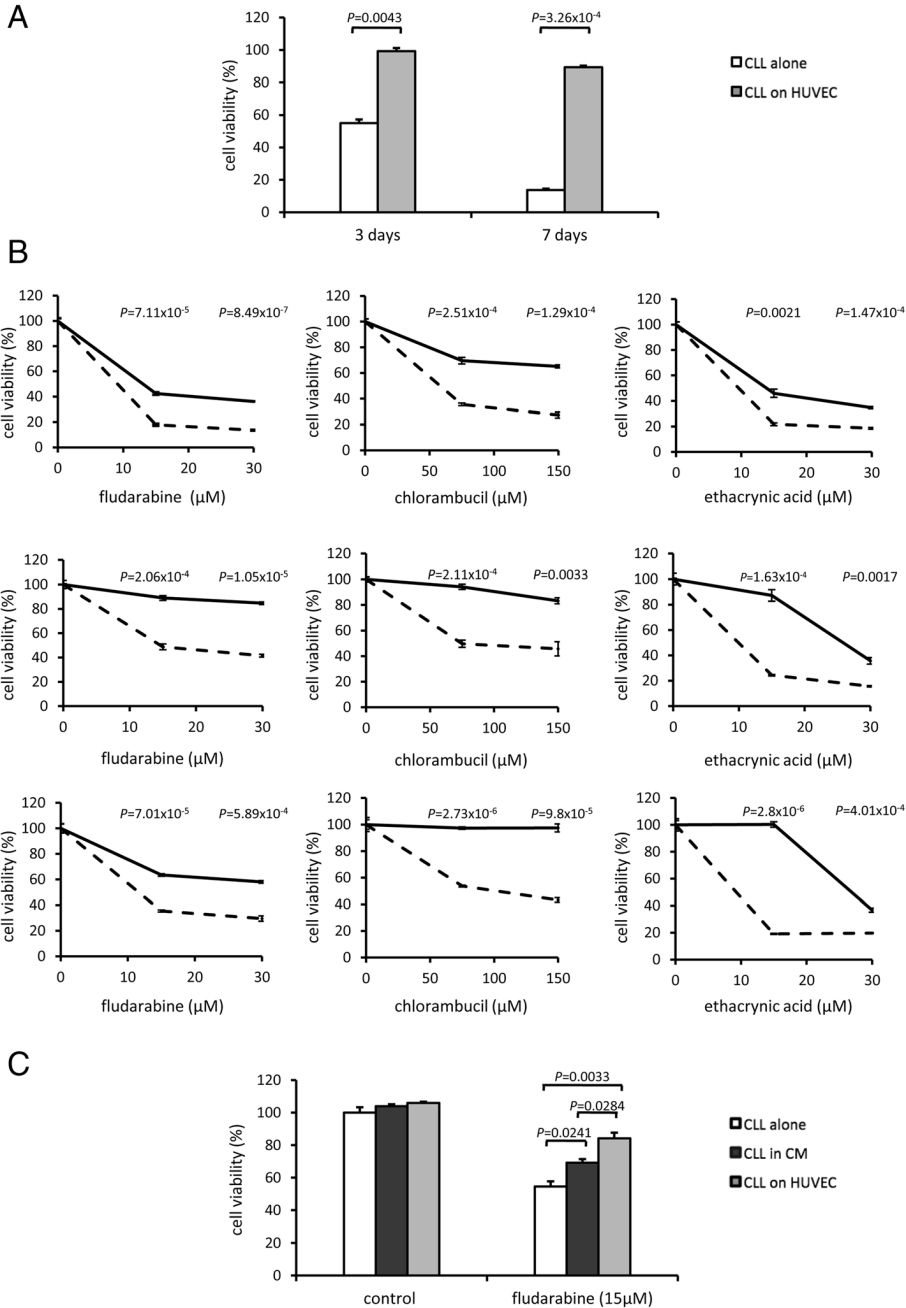
**Human umbilical vein endothelial cells (HUVEC) protect CLL cells from spontaneous and drug-induced cell death. (A)** CLL cells from two patients were cultured alone or on a monolayer of HUVEC for three or seven days. Cell viability was assessed by MTT assay and values were normalized to the uncultured control. Data are from the mean ± SEM of two CLL samples (CLL11 and CLL12) each analysed in triplicate. **(B)** CLL cells from three patients (CLL07, top panel, CLL11, middle panel and CLL12, lower panel) were pre-cultured alone (broken line) or on a HUVEC monolayer (solid line) for 24 hrs. The cells were then cultured under the same conditions in the absence or presence of two different concentrations of fludarabine, chlorambucil or ethacrynic acid for a further 48 hrs. Cell viability was assessed by MTT assay and values were normalized to the respective untreated control for each culture condition. Data are from the mean ± SEM of three independent experiments. The statistical significance of differences in cell viability between control CLL and HUVEC-co- culture CLL is indicated by the *P* values for each drug concentration. **(C)** Conditioned medium (CM) was harvested from a 48 hrs co-culture of CLL cells with HUVECs. CLL cells were pre-cultured in either normal medium or in the CM medium or else on a monolayer of HUVEC for 18 hrs. The cells were then cultured under the same conditions in the presence or absence of fludarabine (15 μM) for a further 48 hrs. Cell viability was assessed by MTT assay and values were normalized to the untreated control. Data show the mean ± SEM from three independent samples.

Recent studies have shown that stromal (accessory) cell-mediated protection of CLL cells from both spontaneous and drug-induced cell death relies on the release of cysteine, an important precursor in glutathione (GSH) synthesis, into the microenvironment and subsequent uptake by CLL cells [33]. Reduced GSH levels are associated with oxidative stress leading to cell death. Since several studies have implicated ID proteins, specifically ID2 and ID3, in the mediation and control of cellular responses to oxidative stress [34-36] and our own data showed enrichment of components of the oxidative stress signalling pathway in MIC-inferred ‘targets’ of ID2/ID3 (see Additional file 6: Table S4), we reasoned that the pro-survival functions of ID2/ID3 in CLL cells might play a role in mediating rescue from cell death by accessory adherent cells. To investigate this, we employed a co-culture system with human umbilical vein vascular endothelial cells (HUVEC) that has previously been shown to rescue CLL cells from spontaneous and fludarabine-induce cell death [37]. Consistent with previous data [37], co-culture with HUVEC rescued CLL cells from both spontaneous (Figure 11A) and fludarabine-induced (Figure 11B) cell death. In addition, we extended these observations to show that HUVEC co-culture also rescued CLL cells from cell death induced by chlorambucil and ethacrynic acid (Figure 11B). To determine whether the protective effect from fludarabine-induced cell death is mediated by direct cell-to-cell contact or by humoral factors, CLL cells were grown in media that had been conditioned by 48 hrs HUVEC-CLL co-culture (Figure 11C). In this ‘double conditioned media’ (CM), the CLL population maintained a significantly higher percentage of viable cells after treatment with 15 μM fludarabine than cells cultured in normal fresh medium. However, this protective effect was significantly less than with cells co-cultured directly with HUVEC (Figure 11C), indicating that the pro-survival functions of HUVEC in response to fludarabine are, as with rescue from spontaneous cell death [37], likely to be mediated by a combination of both humoral and direct heterologous cellular interactions.

**Figure 12:**
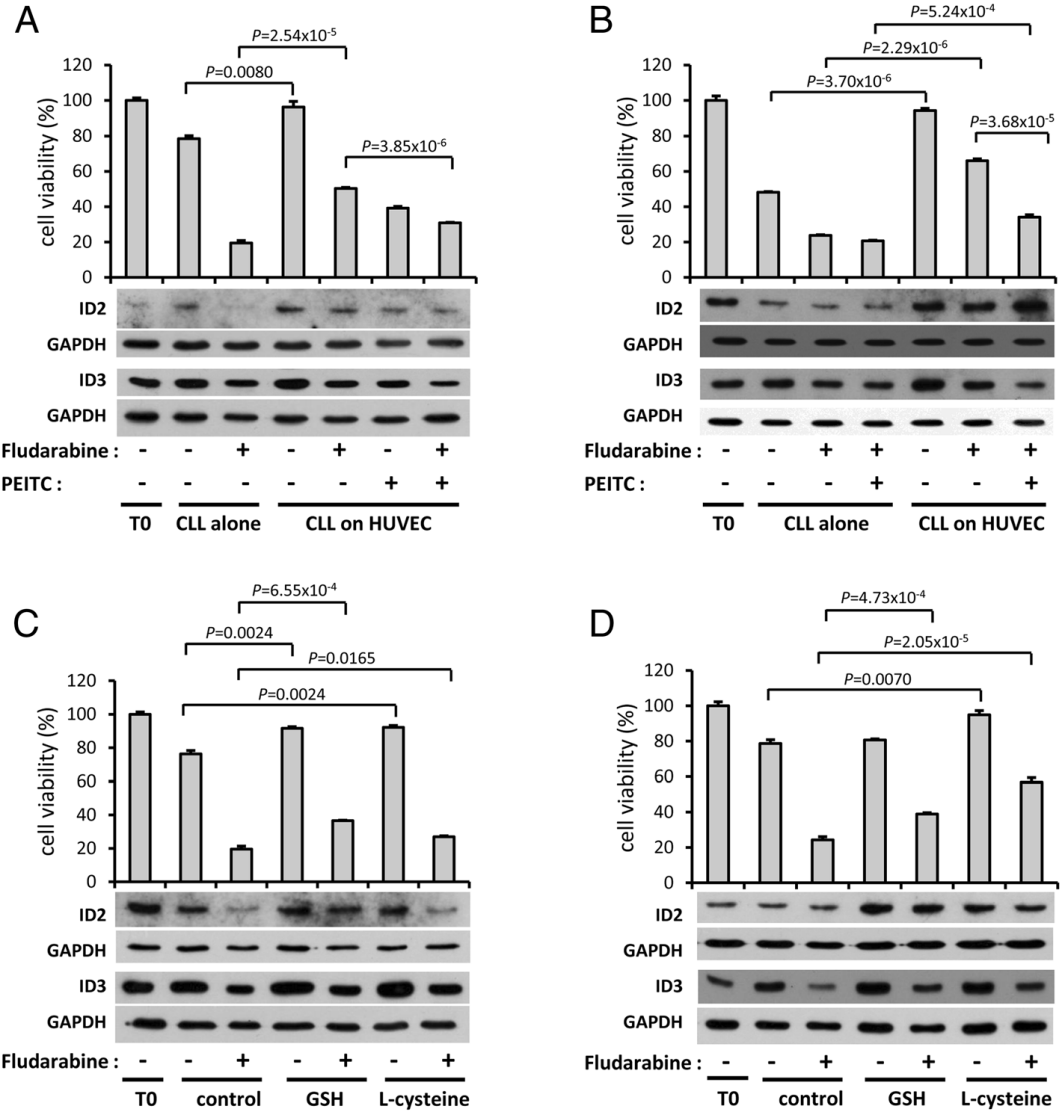
**The effect of HUVEC co-culture and augmentation of CLL glutathione levels on fludarabine-induced cell death and ID2/ID3 protein expression.** CLL12 **(A)** and CLL18 **(B)** cells were pre-cultured alone or on a monolayer of HUVEC for 24 hrs. The cells were then cultured under the same conditions for a further 48 hrs in the absence or presence of fludarabine (20 μM). PEITC (5 μM for **A**, 20 μM for **B**) was added during the last 5 hrs of culture. Cell viability was assessed by MTT assay and values were normalized to the uncultured control (T0). The data show the mean ± SEM of three independent samples. Protein levels of ID2, ID3 and GAPDH were analyzed by western blotting. The original western blot images are shown in Additional file 11: Figure S6. CLL cells from patient CLL12 **(C)** and CLL18 **(D)** were pre-cultured in the absence or presence of GSH (2 mM for **C**, 4 mM for **D**) or L-cysteine (50 μM for **C**, 100 μM for **D**) for 24 hrs. The cells were then cultured for a further 48 hrs in the absence or presence of fludarabine (20 μM). L-cysteine was added to the culture medium daily. Cell viability was assessed by MTT assay and values were normalized to the uncultured control (T0). The data show the mean ± SEM of three independent samples. Protein levels of ID2, ID3 and GAPDH were analyzed by western blotting. The original western blot images are shown in Additional file 11: Figure S6. Quantification of western data by densitometric scanning is shown in Additional file 12: Figure S7.

To evaluate whether GSH release by HUVEC plays a role in mediating this protective effect, the GSH pathway was inhibited by treatment with PEITC, a thiol conjugate that depletes intracellular GSH levels [38] during HUVEC-CLL co-cultures. As shown in Figure 12A & B (and see Additional file 11: Figure S6; Additional file 12: Figure S7), co-culture with HUVEC increased both ID2 and ID3 protein levels in two CLL samples examined, an effect which correlated with rescue from spontaneous and fludarabine-induced cell death. PEITC treatment partially overcame the rescue from fludarabine-induced cell death imparted by HUVEC co-culture, and this was accompanied by decreased ID2 and ID3 protein expression. Specifically, For CLL12 (Figure 12A and see Additional file 11: Figure S6) a comparison between the most relevant control of fludarabine-treated cells cultured on HUVECs (lane 5) with the same cells treated additionally with PEITC (lane 7) showed a clear reduction (approximately two-fold) in both ID2 and ID3 in the latter (see densitometric data in Additional file 12: Figure S7). The same comparison for CLL18 (lane 6 vs lane 7 in Figure 12B) similarly revealed a small (albeit insignificant) reduction in ID2, but the reduction in ID3 was very comparable to that seen for CLL12. Hence, even for CLL18, which was one of the most ‘extreme’, fludarabine-resistant samples, there was a reduction in expression of at least one of the ID proteins following partial blockade of GSH signalling in HUVEC co-cultures following treatment with fludarabine. To confirm that GSH release is a probable mechanism through which HUVEC co-culture promotes CLL survival and that it affects modulation of ID protein expression, CLL cells were treated with GSH or L-cysteine. Consistent with the report of Zhang et al. [33] we found that both compounds protect from spontaneous and fludarabine-induced cell death (Figure 12C & D and see Additional file 12: Figure S7). In addition, both GSH and L-cysteine mimic the presence of HUVEC by commensurately increasing ID2 and ID3 protein levels.

## Discussion

Previous studies have shown that ID2 and ID3 are crucial mediators of B-lymphocyte development and are also involved in the regulation of B-cell viability (reviewed in [4,39]), functioning to promote either B-cell death or B-cell survival, depending on the particular context. In CLL, our bioinformatics analysis showed that the expression profiles of *ID2* and *ID3* are associated with distinct pathobiological features of this disease. ID2 expression was down-regulated in CLL versus normal B cells in most microarray datasets. Consistent with this, high expression of *ID2* was associated with a more favourable clinical outcome. By contrast, *ID3* expression was consistently up-regulated in CLL versus normal B cells but exhibited no significant association with clinical end-points, at least when analysed in available datasets. Both *ID* genes displayed a distinct expression profile amongst the different molecular sub-types of CLL that have been defined previously [25] and MIC analysis showed that the two *ID* genes are coupled to gene regulatory networks that are largely non-overlapping in CLL. However, gene set and pathway enrichment analysis suggested that both *ID* genes function in many of the same biological processes and pathways by regulating the expression (directly or indirectly) of a distinct set of target genes. This analysis invoked regulation of apoptosis/cell death in which both *ID* genes play a major role in CLL. It should be noted that because of the non-directional nature of MIC-inferred regulatory interactions, not all of the genes identified by MIC analysis are necessarily regulated by *ID2*/*ID3* (rather than *vice versa*). Nonetheless, given the large number of apoptosis genes that were identified from pathway enrichment analysis (see Figure 4), it is plausible that ID2 and ID3 each regulate the expression of a sizable number of genes (both pro- and anti-apoptotic) involved in cell survival of CLL.

Recent whole-genome and exome sequencing of CLL has revealed that, in contrast to the reported high frequency of recurrent ID3 mutations observed in Burkitt lymphoma and (less commonly) in some other B-lymphoma types [11-13], mutations in ID genes do not occur at a significant frequency in CLL [18] (and references therein). Indeed, of the several hundred CLL cases so far sequenced in different laboratories, only a single instance of a mutated ID gene has so far been reported (a missense mutation - E48V) affecting ID2 [40], the functional significance of which is unknown. Consistent with the bioinformatics analysis (above), our data show that, at the protein level, the expression of ID2 and ID3 is extremely heterogeneous amongst different CLLs and, from siRNA knock-down experiments, both proteins appear to perform pro-survival functions in both spontaneous and drug-induced cell death in this leukemic cell type. Although based on a very small cohort of CLL samples, we also observed a possible association between low ID3 protein expression (before drug treatment) and *in vitro* drug resistance for both fludarabine and chlorambucil. On exposure to these drugs, this was reflected by a pattern of down-regulation of ID3 expression in the more drug-sensitive and up-regulation in the most drug-resistant samples, consistent with the pro-survival function of this ID protein. Albeit with different kinetics, chlorambucil also elicited down-regulation of at least one of the two ID proteins in chlorambucil-sensitive CLLs and up-regulation of both ID proteins in the CLL that was most resistant to this drug. Finally, the drug, ethacrynic acid, which in contrast to fludarabine and chlorambucil, acts at least in part through inhibition of the Wnt/β-catenin signalling pathway [30] that is also known to be a key regulator of *ID* gene expression [2,3] (and was also inferred from pathway enrichment analysis of *ID2* MIC ‘targets’ in CLL – see Additional file 6: Table S4). Perhaps unsurprisingly, ethacrynic acid elicited dramatic changes in ID2/ID3 protein expression that were characterised by marked up-regulation of ID2/ID3 levels in those CLLs that were the most resistant to this drug, again consistent with a pro-survival function for the ID2/ID3 proteins in the ethacrynic acid cytotoxic response.

We also noted a possible association between high ID3 protein levels and resistance to spontaneous cell death in the absence of drug treatment, again consistent with a pro-survival function for this ID protein, although this observation should again be interpreted with caution since the analysis was based on a very small cohort of CLLs. However, these findings are in accord with published microarray gene expression studies; datamining reveals that *ID3* mRNA levels are significantly higher in the IGHV-mutated subset of CLL [41], that is reportedly more resistant to *in vitro* spontaneous cell death, than IGHV-unmutated CLLs [42]. Moreover, low *ID3* mRNA expression is part of a distinct gene expression signature associated with ATM-mutated CLL [43]. ATM-mutated CLL (low *ID3*) define a sub-group of patients with an unfavourable clinical course [43] and IGHV-mutated CLL (high *ID3*) define a more favourable prognostic sub-group (reviewed in [44]).

Knock-down of ID2/ID3 expression dramatically reduced cell viability in the MEC1 cell line model. But as with the ID expression data, the effect of siRNA knock-down in primary CLL was heterogeneous. A statistically significant effect of ID2/ID3 knock-down on cell viability in the absence of fludarabine treatment was observed in three of the four CLLs examined. The exception (CLL18) expressed the lowest levels of ID2 and ID3, consistent with the observed diminished effect of ID knock-down in these cells. Following fludarabine treatment, the effect of ID knock-down on cell viability was even more heterogeneous, reaching statistical significance in only one CLL, but with an appreciable effect in a further two.

Recent studies have shown that expression of the E-protein bHLH transcription factor, E2A/TCF3, that is one of the key targets of ID proteins, is elevated relative to normal B cell subsets in CLL and also promotes cell survival [45]; *E2A* mRNA knock-down leads to reduced CLL cell viability [45]. A similar effect of *E2A* mRNA knock-down has also been described in prostate cancer cells where it was shown to cause down-regulation of ID gene expression [46]. Since we have shown that loss of ID2/ID3 expression leads to loss of viability in CLL cells, the cell death reported to accompany loss of E2A expression in CLL cells [45] may well be mediated via loss of ‘downstream’ ID protein expression.

The interaction between CLL cells and the bone marrow/lymph node stromal cell environment *in vivo* is known to profoundly affect CLL cell viability and drug sensitivity, and this can be recapitulated *in vitro* by co-culture with accessory bone marrow stromal cells [47] or with vascular endothelial cells [37,48]. We found that depletion of GSH using PEITC abrogated HUVEC-mediated rescue of CLL cells from both spontaneous and fludarabine-induced cell death, implicating a redox-dependent pro-survival mechanism imparted by HUVEC cells, similar to that reported for bone marrow stromal cells [33]. Although off-target effects of PEITC, at the concentrations used in our study, have not been noted in previous studies on CLL [33,38] this cannot however be completely ruled out. Consistent with recently published gene expression microarray data [37], co-culture with HUVEC cells led to up-regulation of ID2 and or ID3 protein expression in CLL cells, an affect that was modulated by PEITC depletion of GSH or by direct addition of GSH or L-cysteine to an extent commensurate with rescue from cell death. These observations are in accord with the pathway enrichment analysis of MIC-inferred regulatory interactions (see Additional file 6: Table S4) which identified the oxidative stress response as a pathway shared by both ID2 and ID3. Although we did not directly determine intracellular GSH levels in our study, the role of GSH in survival of CLL (either in isolation or co-culture with accessory cells) has previously been established by other laboratories from direct measurement of intracellular GSH [33]. It should be noted that the data in this part of our investigation was based on analysis of only two CLLs. However, given that these CLLs were quite different (one sensitive, the other highly resistant to fludarabine) and both gave broadly consistent results, the data could be considered to be ‘representative’. With this caveat in extrapolating the findings to CLL too generally, our data support a model in which HUVEC co-culture imparts its protective effect on CLL cells at least in part by increasing intracellular GSH levels, which in turn leads to increased expression of the redox-responsive pro-survival ID2 and ID3 proteins.

## Conclusions

In summary, we have undertaken a comprehensive datamining investigation of CLL using available gene expression microarray data to show that the expression profiles of *ID2* and *ID3* are associated with distinct pathobiological features of this disease and both are strongly implicated in regulating cell death/survival in CLL cells. Experimental evidence supported a pro-survival role for both ID proteins in CLL and was extended to show that, in a more physiologically-relevant *in vitro* co-culture system, vascular endothelial cells rescue CLL cells from spontaneous and cytotoxic drug-induced cell death via an ID protein-coupled redox-dependent mechanism. Future studies might usefully explore the precise molecular mechanisms through which ID proteins are involved in disease pathogenesis and treatment response in CLL with a view to identifying candidate targets for therapeutic intervention in this disease.

## Materials and methods

### Ethics statement

The study protocol including consent procedures was approved by the UK local NHS Ethics Committee (protocol reference: 08/H0302/90). Peripheral blood samples were obtained from CLL patients, together with ‘anonymised’ patient data after informed written consent in accordance with the principles expressed in the Declaration of Helsinki. All records (including signed consent forms) were maintained in a secure database at the Ipswich Hospital NHS Trust, Suffolk, UK.

### Datamining of microarray gene expression data

For analysis of *ID2*/*ID3* gene expression in CLL versus normal B cells, normalized microarray gene expression datasets were obtained from the NCBI Gene Expression Omnibus database [49]. Samples representing normal B and CLL cells were curated from each dataset and, after log_2_ transformation of expression values, differential expression was analysed using the limma package [50] in Bioconductor R. *P* values for the significance of differential expression were corrected for false discovery rate [51].

Two microarray datasets with publicly available clinical follow-up data were fRMA-normalised [52] and downloaded from *InSilico DB* [53]: GSE39671 [54] with annotation data on time to first treatment for 130 patients, and GSE22762 [55] with annotation data on both time to first treatment and survival time for 107 patients. Kaplan-Meier plots were constructed using GenePattern [56] by partitioning samples according to *ID* gene expression into *ID2*/*ID3*-high (upper 50%) and *ID2*/*ID3*-low (lower 50%) patient groups. The statistical significance of differences in Kaplan-Meier plots was determined by log-rank test.

Consensus clustering analysis was performed essentially as described previously [25] except that a composite dataset comprised of 871 CLLs from 14 individual datasets (omitting GSE15777) was used. Briefly, CLL samples from each dataset were curated in *InSilico DB* [53] and the fRMA-normalised datasets were downloaded and merged using the ‘COMBAT’ algorithm with the inSilicoMerging R/Bioconductor package [57]. The combined dataset was marker center-normalized and analysed with the ‘ConsensusClusterPlus’ package in R/Bioconductor [58] using Euclidean distance and Ward2 agglomerative methods with 1000 iterations. The optimum number of cluster groups (seven) was ascertained from the delta area plot where there was minimal relative decrease in the consensus cumulative distribution function (CDF). Gene signatures representing each cluster group (gene sets significantly up-regulated in each cluster group) were generated using the GenePattern *limma* package [50,56] applying a Bonferroni-corrected *P* value threshold of 0.01 and were analysed for significant overlap with KEGG pathway and Oncogenic signatures databases using the GSEA on-line database [59].

To identify candidate target genes that are regulated by ID2/ID3 in CLL, we employed the ‘maximal information-based nonparametric exploration’ (MINE) statistics package in Bioconductor, R [26] to compute maximum information coefficient (MIC) scores and other metrics as a measure of the statistical dependency between expression of *ID2*/*ID3* and all other genes in the composite 871 CLL microarray dataset (above). A threshold cut-off MIC score corresponding to a Bonferroni-corrected *P* value (calculated using 3×10^7^ permutations) of 0.01 was applied to identify the most statistically significant candidate ‘target’ genes. The resulting gene lists were analysed for over-representation of Gene Ontology (biological process) and pathway gene sets using the GeneCodis database [60]. Candidate apoptosis target genes were further investigated for protein-protein interactions using the ‘String’ (v9.1) database [28] and for literature-validated regulatory interactions using the ‘Unified Human Interactome’ (UniHI) database [29]. A network graph was constructed and visualised using Cytoscape v2.8 [61].

### CLL patients, cell isolation and culture conditions

Peripheral blood samples from 14 CLL patients were obtained from patients attending the Hematology out-patient clinic at Ipswich Hospital NHS Trust (Suffolk, UK). Patients were selected having a white cell count of >45×10^9^/L in order to ensure a high representation of CLL cells. Patient characteristics are listed in Additional file 7: Table S5. Peripheral blood mononuclear cells were isolated by density gradient centrifugation using Ficoll-Paque PLUS (GE Healthcare, Little Chalfort, UK) according to the manufacturer’s protocol. The MEC1 cell line [31] (DMSZ, Braunschweig, Germany) and primary CLL cells were cultured in IMDM supplemented with 10% fetal bovine serum (FBS) and 45 μg/ml gentamicin (all from PAA, Pasching, Austria) at 37°C and 5% CO_2_ in a humidified incubator. Primary CLL cells were seeded at a density of 1.5-5×10^6^ cells/ml, while MEC1 cells were maintained at a cell density between 0.5-1×10^6^ cells/ml.

Human umbilical vein endothelial cells (HUVEC, TCS Cellworks, Buckingham, UK) were cultured in human large vessel endothelial cell growth medium (TCS Cellworks) on poly-lysine-coated tissue culture flasks. For co-culture experiments, HUVECs were seeded at 60% density and incubated for 24 hrs before the medium was removed and CLL cells were seeded on top of the monolayer at 1.5×10^6^ cells/ml in IMDM/10% FBS. Cells were maintained under these co-culture conditions for 24 hrs, prior to drug addition and then cultured in the presence of drugs for another 48 hrs. The ‘double conditioned medium’ (CM), was harvested from a co-culture of HUVEC and CLL cells after 48 hrs, filter-sterilized and stored at 4°C until required. Glutathione (GSH), Phenethyl-isothiocyanate (PEITC), L-cysteine, fludarabine, chlorambucil and ethacrynic acid were purchased from Sigma-Aldrich (St-Louis, MO, USA).

### Lentivirus production and infection of MEC1 cells

Lentiviral vectors encoding siRNA targeting either control or the *ID2* and *ID3* genes were purchased from Applied Biological Materials (ABM) Inc. (Richmond, BC, Canada). The vector backbone (piLenti-siRNA-GFP) contains convergent U6 and H1 promoters producing double-stranded siRNA molecules. The sequences targeted by these siRNAs were as follows:

- *ID2R*-siRNA: 5’-TGTGGACGACCCGATGAGC-3’,
- *ID2Y*-siRNA: 5’-ATCGACTACATCTTGGACCTGCAGATCGC-3’,
- *ID2G*-siRNA: 5’-CCCACTATTGTCAGCCTGCATCACCAGAG-3’,
- *ID2B*-siRNA: 5’-TCTGAGTTAATGTCAAATGACAGCAAAGC-3’,
- *ID3R-*siRNA: 5’-ACTCAGCTTAGCCAGGTGGAAATCCTACA-3’,
- *ID3Y*-siRNA: 5’-ATCGACTACATTCTCGACCTGCAGGTAGT-3’,
- *ID3G*-siRNA: 5’-ACCTTCCCATCCAGACAGCCGAGCTCACT-3’,
- *ID3B*-siRNA: 5’-CCGGAACTTGTCATCTCCAACGACAAAAG-3’,
- Negative control siRNA: 5’-GGGTGAACTCACGTCAGAA-3’.

The second generation packaging plasmids psPax2 and pMD2.G were purchased from Addgene (deposited by Prof. Didier Trono, Lausanne, Switzerland). Human embryonic kidney (HEK 293 T) cells were cotransfected with 2 μg siRNA lentiviral expression vector together with 1.3 μg psPax2 and 0.7 μg pMD2.G using the calcium phosphate method and then used for a 48 hr co-culture with 5×10^5^ MEC1 cells followed by selection for 14 days with puromycin (1.5 μg/ml). Stable pools of transduced MEC1 cells were then expanded in IMDM/10% FBS without puromycin.

### siRNA-mediated knock-down of ID protein expression

siRNA transfection of primary CLL cells was performed using a HiPerfect transfection kit (Qiagen, Hilden, Germany), according to the manufacturer’s guidelines. Briefly, 1×10^6^ CLL cells were seeded in 200 μl IMDM/10%FBS and transfected with 60nM siRNA (final concentration in 600 μl) and 5 μl HiPerfect transfection reagent in 100 μl IMDM. After 6 hrs incubation, 300 μl of fresh IMDM/10%FCS containing 45 μg/ml gentamicin were added. Knockdown efficiency was assessed by western blotting after 72 hrs. Pre-designed, chemically-modified, siRNA oligoribonucleotides (Stealth™) targeting *ID2* or *ID3*, as well as a negative control (Stealth™ medium GC Duplex) were purchased from Invitrogen (Carlsbad, CA, USA). The sequences of the *ID* siRNAs were as follows:

- *ID2*: 5’- ACGTCATCGACTACATCTTGGACCT-3’;
- *ID3*: 5’- GGCACTCAGCTTAGCCAGGTGGAAA-3’

### Cell viability assay

Cell viability was assessed using the 3-(4,5-dimethilthiazol-2yl)-2,5-diphenyl tetrazolium bromide (MTT) assay. MEC1 cells were incubated with 0.5 mg/ml MTT for 2 hrs at 37°C, 5% CO_2_, while primary CLL cells required 4 hrs of incubation. Cells were then harvested and recovered by centrifugation and incubated in 100–200 μl dimethylsulphoxide at 37°C for 20 minutes before optical density was recorded in a microplate spectrophotometer at 560 nm. The percentage of cell viability assessed by MTT assay was used to determine IC_50_ concentrations from dose–response curves at 72 hrs following treatment with 4 different drug concentrations.

### Western blotting

Cells were lysed, electrophoresed on polyacrylamide (15%)-SDS gels followed by western blotting, essentially as described previously [62]. After incubation with primary rabbit polyclonal antibodies against either ID2 (sc-489) or ID3 (sc-490) (Santa Cruz Biotechnology, Dallas, TX, USA), membranes were washed in 0.05% Tween-20 in TBS and incubated for 1 hr with polyclonal goat horseradish peroxidase–conjugated secondary antibody (AbCam, Cambridge, UK). Following extensive washes, bound antibodies were visualized by enhanced chemiluminescence (ECL, Millipore, Billerica, MA, USA) on X-ray films. Membranes were then stripped by incubation with stripping buffer (25 mM glycine pH2, 1% SDS) for 30 minutes, washed and reprobed with primary rabbit polyclonal antibody against GAPDH (Sigma-Aldrich), followed by secondary antibody detection as described above. Protein bands on western blot films were quantified by densitometric scanning and analysis using ‘ImageJ’ software. Heatmap images were created using the ‘HeatMapViewer’ module in the GenePattern software suite [56].

### *Ad hoc* statistical analysis

For MTT assay data, continuous variables were compared using the Student’s *t*-test while correlation between continuous variables was performed by determination of Pearson’s correlation coefficient using the ‘VassarStats’ online statistical calculator [63]. All statistical tests were two-sided. Boxplots were generated by using the ‘BoxPlotR’ online tool [64]. Statistical analysis of gene overlap by hypergeometric distribution was performed using the ‘phyper’ algorithm in Bioconductor R.

## Additional files

Additional file 1: Figure S1. Kaplan-Meier plots showing the relation between *ID3* expression and clinical outcome in CLL. A: analysis of time to first treatment for GSE39671 dataset; B: analysis of time to first treatment for GSE22762 dataset; C: analysis of survival time for GSE22762 dataset. For each dataset, patients were grouped according to high (red line) and low (blue line) *ID3* expression. The significance of the difference in clinical end-point between high and low *ID3* expression patient groups was determined by log rank test. Additional file 2: Figure S2. Performance of consensus clustering showing optimum partitioning of seven CLL sub-types. A: Delta area plot showing increase in area under the consensus cumulative distribution function for different numbers of sub-groups (‘k’ on x axis); B: Heatmap representation of the consensus matrix for k = 7 sub-groups. Additional file 3: Table S1. KEGG pathway enrichment for molecular sub-types identified by consensus clustering. The top 20 pathways that significantly overlap with the gene signature defining each of the 7 molecular sub-type (cluster groups – clu) are shown. Additional file 4: Table S2. Oncogenic signature enrichment for molecular sub-types identified by consensus clustering. The top 20 oncogenic signatures that significantly overlap with the gene signatures defining each of the 7 molecular sub-type (cluster groups – clu) are shown. Additional file 5: Table S3. MIC scores and other metrics from MINE investigation of *ID2*/*ID3*-coupled genes in CLL. Gene symbols that are common between the *ID2* and *ID3* lists are highlighted in yellow. Additional file 6: Table S4. Gene Ontology and pathway enrichment analysis of ID2 and ID3 candidate targets inferred from MIC analysis. Only the top-ranked 25 Gene Ontology terms for biological process are shown. Pathways that are common to both ID2 and ID3 MIC-inferred targets are highlighted in yellow. Additional file 7: Table S5. Description of CLL patient characteristics. Additional file 8: Figure S3. Original scanned images of western blot analysis of ID protein expression levels in primary CLL. The images shown were used to compile Figure 5A in the main manuscript. The order of CLL samples in the left and right-hand panels corresponds to that in Figure 5A. An indicative size marker scale in kDa is shown together with identities of the 36 kDa GAPDH and ID2/ID3 protein bands. The latter were verified in independent siRNA knock-down and transfection-over-expression experiments. Note that only the relevant sections of blots were re-probed with antibody for GAPDH. Additional file 9: Figure S4. Quantification of western blot analysis of ID protein expression levels in primary CLL. Band intensities of the western data shown in Figure 5A were quantified by densitometric scanning using ‘ImageJ’ software. Data for raw band intensities is shown. Normalised band intensity data is presented in Figure 5B of the main manuscript. Additional file 10: Figure S5. Original scanned images of western blot analysis of ID protein expression in siRNA knock-down experiments. The images shown were used to compile Figure 10A&C in the main manuscript. The order of samples for the analysis of MEC1 cells is the same as in Figure 10. An indicative size marker scale in kDa is shown together with identities of the 36 kDa GAPDH and ID2/ID3 protein bands. The latter were verified in independent siRNA knock-down and transfection-over-expression experiments. Note that only the relevant sections of blots were re-probed with antibody for GAPDH. Additional file 11: Figure S6. Original scanned images of western blot analysis of ID protein expression in HUVEC co-culture experiments. The images shown in panels A-D were used to compile Figure 12 in the main manuscript. The order of CLL samples in each panel (A-D) corresponds to that in Figure 12. An indicative size marker scale in kDa is shown together with identities of the 36 kDa GAPDH and ID2/ID3 protein bands. The latter were verified in independent siRNA knock-down and transfection-over-expression experiments. Note that only the relevant sections of blots were re-probed with antibody for GAPDH. Additional file 12: Figure S7. Quantification of western blot analysis of ID protein expression levels in CLL cells following manipulation of intracellular GSH levels. Band intensities of the western data shown in Figure 12 of the main manuscript were quantified by densitometric scanning using ‘ImageJ’ software. Data were normalized to the GAPDH loading control and, for each ID protein, expressed as fold-change (relative band intensity) relative to the uncultured control (T0).

## Abbreviations

CDF: Consensus cumulative distribution
CLL: B-cell chronic lymphocytic leukemia
CM: Double conditioned medium
fRMA: Frozen robust multiarray analysis
GSEA: Gene set enrichment analysis
GSH: Glutathione
HUVEC: Human umbilical vein vascular endothelial cells
IGHV: Immunoglobulin heavy chain variable region
MIC: Maximum information coefficient
MINE: Maximal information-based nonparametric exploration
MTT: 3-(4,5-dimethilthiazol-2yl)-2,5-diphenyl tetrazolium bromide
PEITC: Phenethyl-isothiocyanate
UniHI: Unified Human Interactome
