# Supplementary figures and images for: ID helix-loop-helix proteins as determinants of cell survival in B-cell chronic lymphocytic leukemia cells in vitro
Source: Mol Cancer. 2015 Feb 3;14(1):30. doi: 10.1186/s12943-014-0286-9 (PMC4320821; doi:10.1186/s12943-014-0286-9)

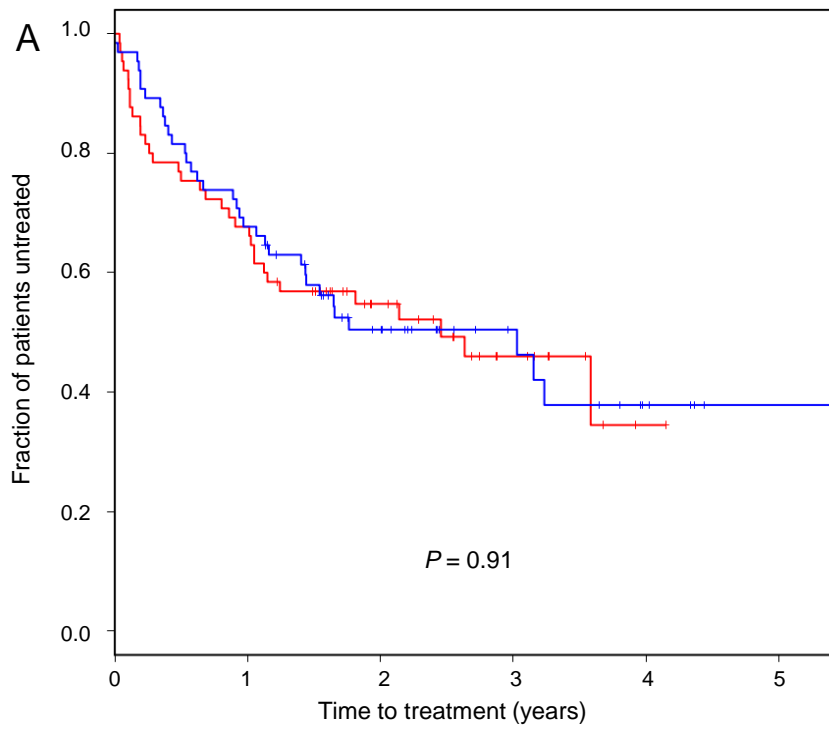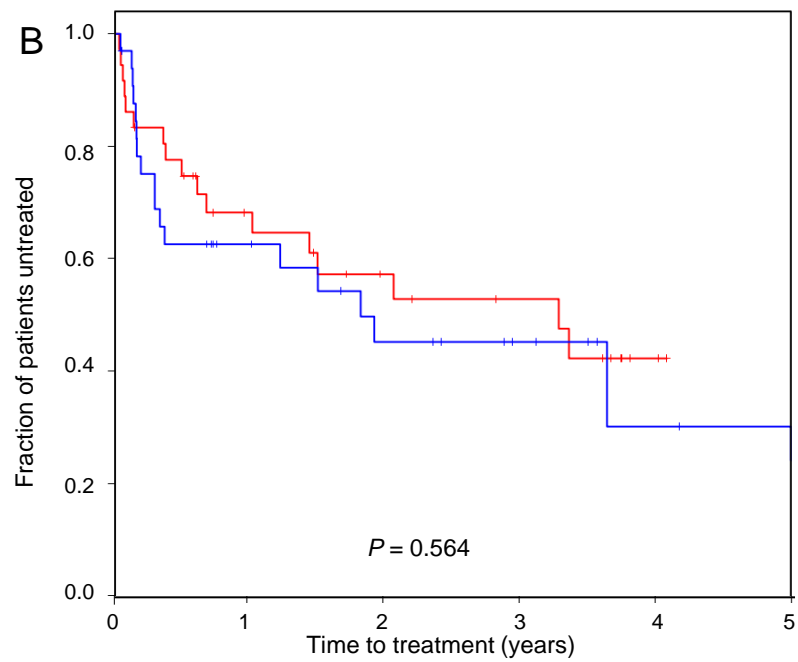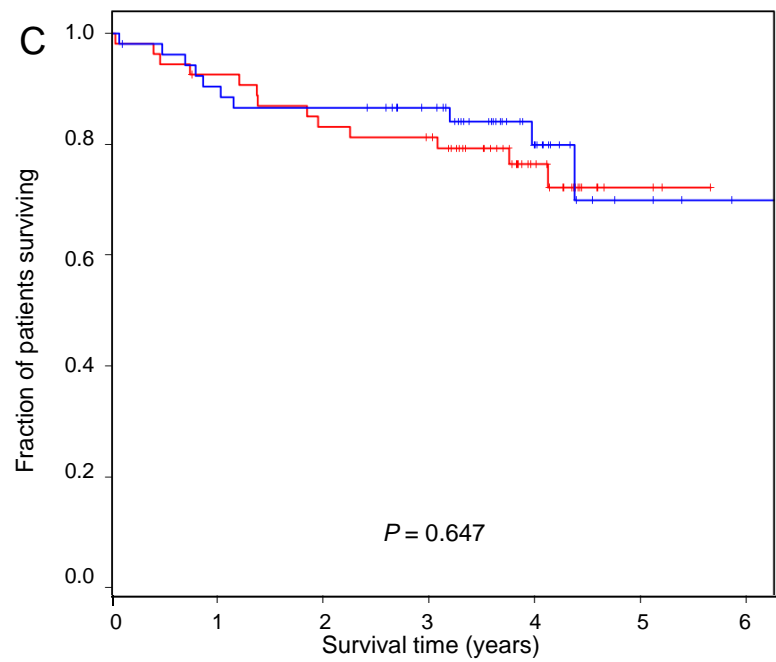

Supplement: Additional file 1: Figure S1. — Kaplan-Meier plots showing the relation between ID3 expression and clinical outcome in CLL. A: analysis of time to first treatment for GSE39671 dataset; B: analysis of time to first treatment for GSE22762 dataset; C: analysis of survival time for GSE22762 dataset. For each dataset, patients were grouped according to high (red line) and low (blue line) ID3 expression. The significance of the difference in clinical end-point between high and low ID3 expression patient groups was determined by log rank test. [file 12943_2014_286_MOESM1_ESM.pdf]

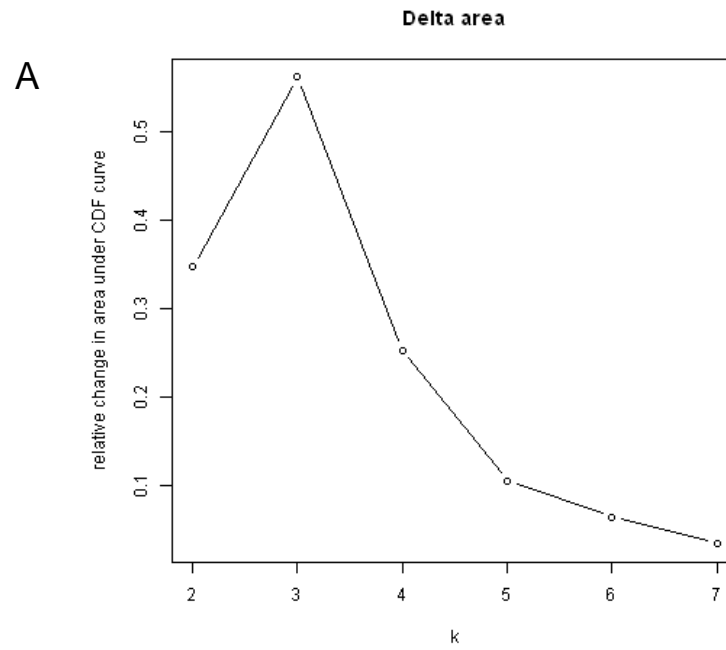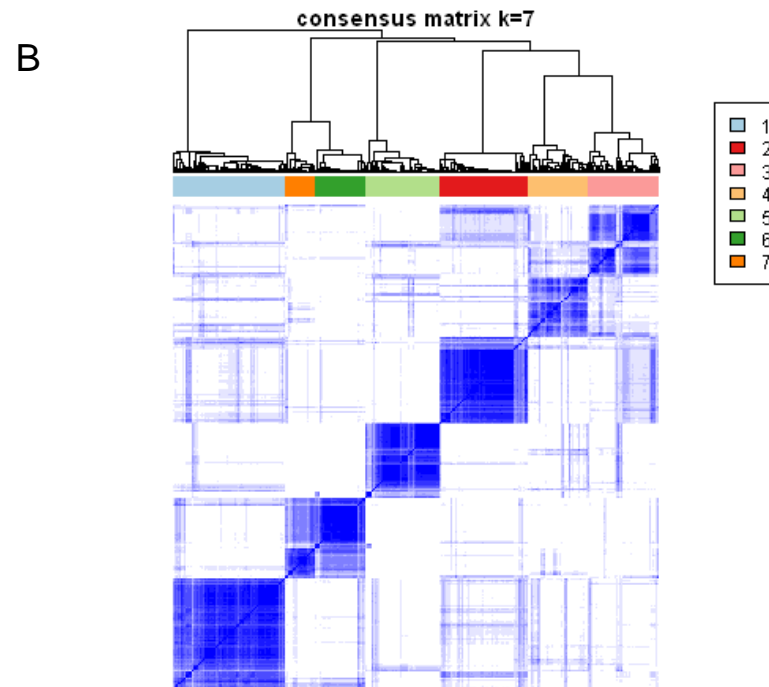

Supplement: Additional file 2: Figure S2. — Performance of consensus clustering showing optimum partitioning of seven CLL sub-types. A: Delta area plot showing increase in area under the consensus cumulative distribution function for different numbers of sub-groups (‘k’ on x axis); B: Heatmap representation of the consensus matrix for k = 7 sub-groups. [file 12943_2014_286_MOESM2_ESM.pdf]

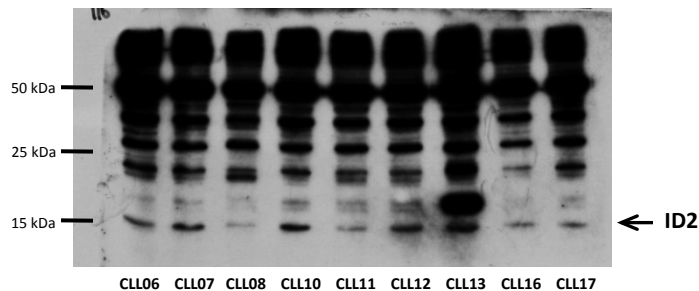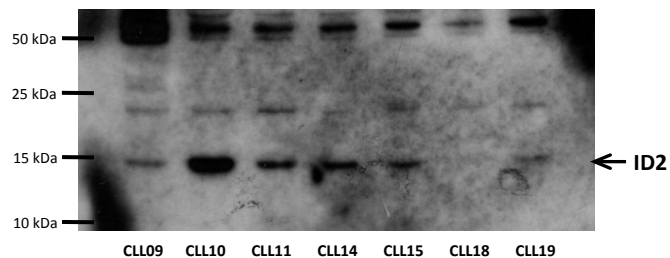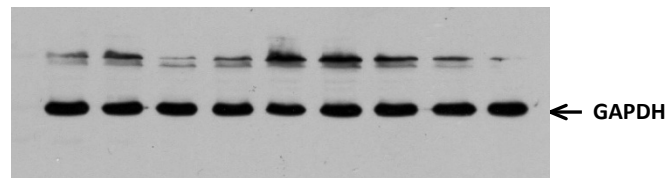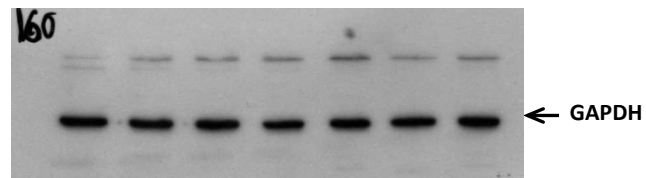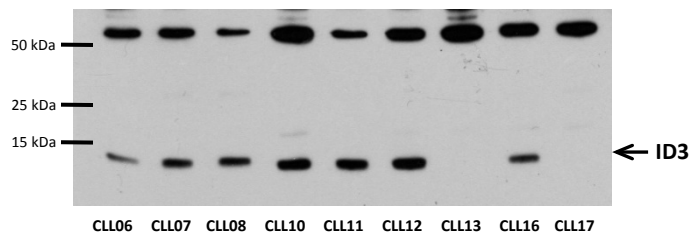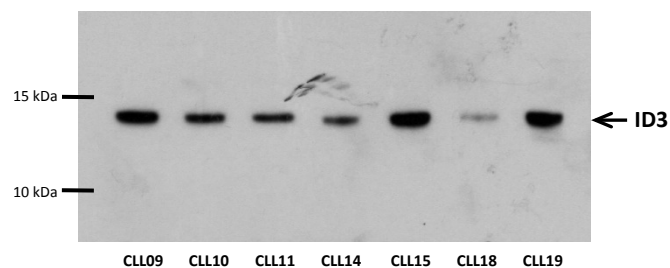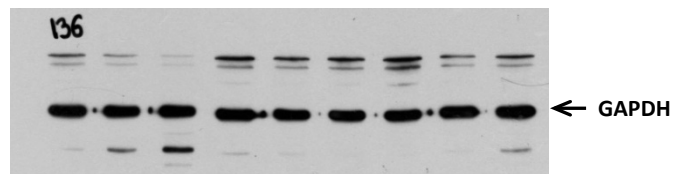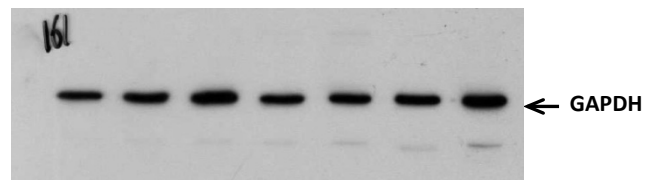

Supplement: Additional file 8: Figure S3. — Original scanned images of western blot analysis of ID protein expression levels in primary CLL. The images shown were used to compile Figure 5A in the main manuscript. The order of CLL samples in the left and right-hand panels corresponds to that in Figure 5A. An indicative size marker scale in kDa is shown together with identities of the 36 kDa GAPDH and ID2/ID3 protein bands. The latter were verified in independent siRNA knock-down and transfection-over-expression experiments. Note that only the relevant sections of blots were re-probed with antibody for GAPDH. [file 12943_2014_286_MOESM8_ESM.pdf]

**ID2**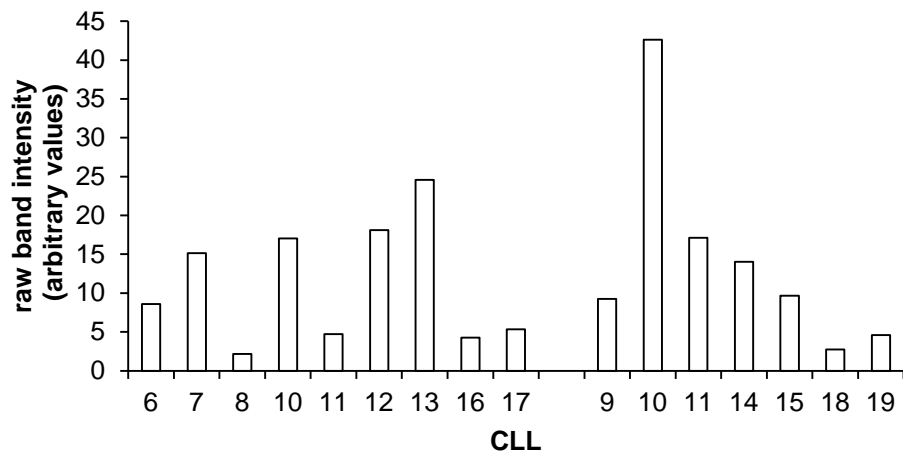**ID3**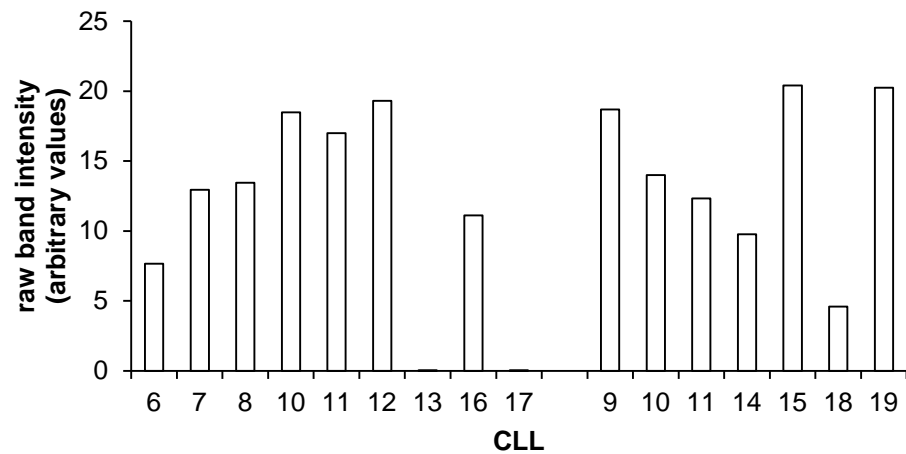**GAPDH (ID2)**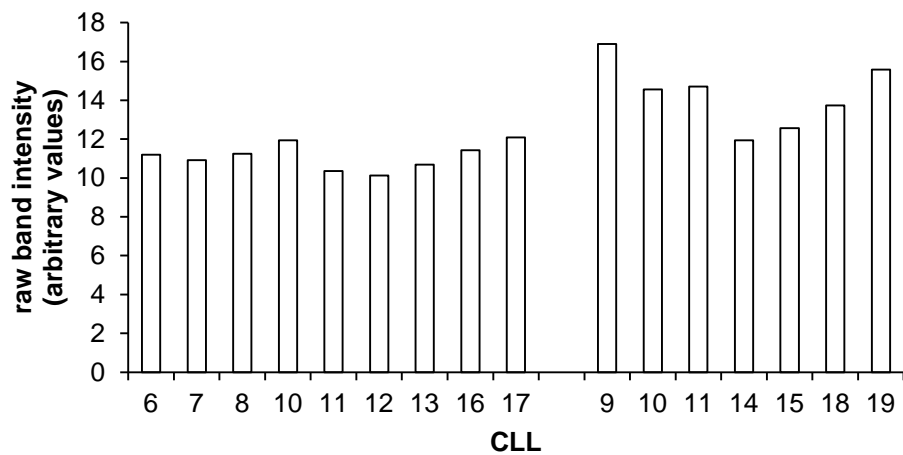**GAPDH (ID3)**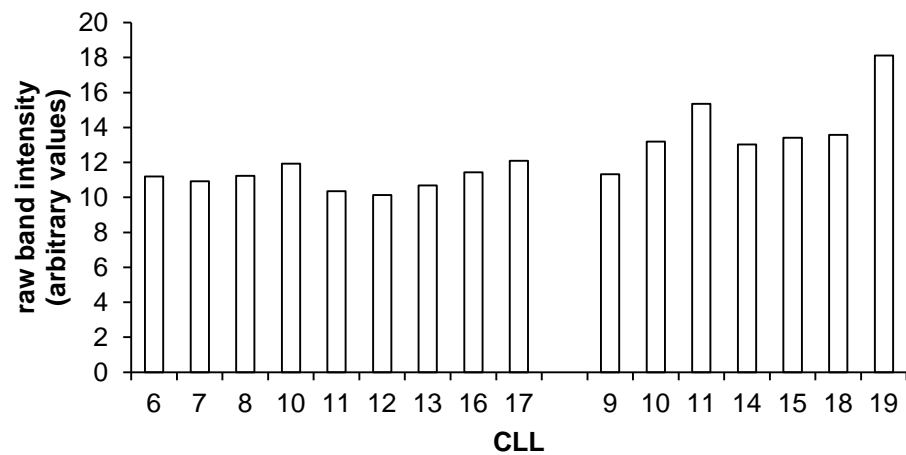

Supplement: Additional file 9: Figure S4. — Quantification of western blot analysis of ID protein expression levels in primary CLL. Band intensities of the western data shown in Figure 5A were quantified by densitometric scanning using ‘ImageJ’ software. Data for raw band intensities is shown. Normalised band intensity data is presented in Figure 5B of the main manuscript. [file 12943_2014_286_MOESM9_ESM.pdf]

**MEC1  
cells**

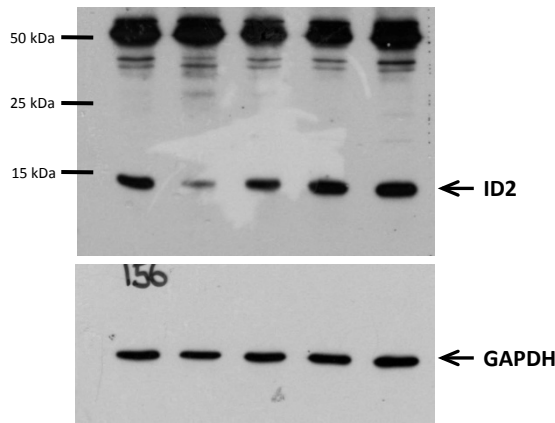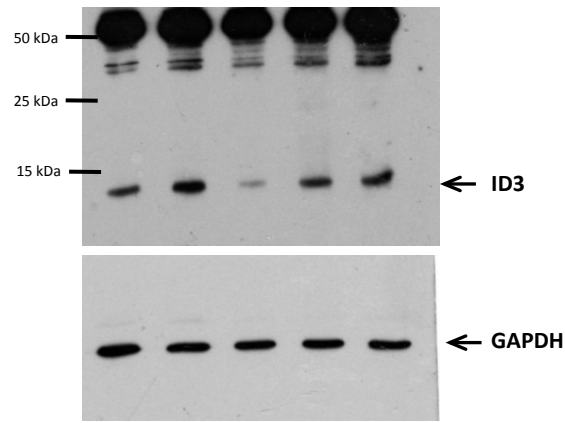

**CLL  
cells**

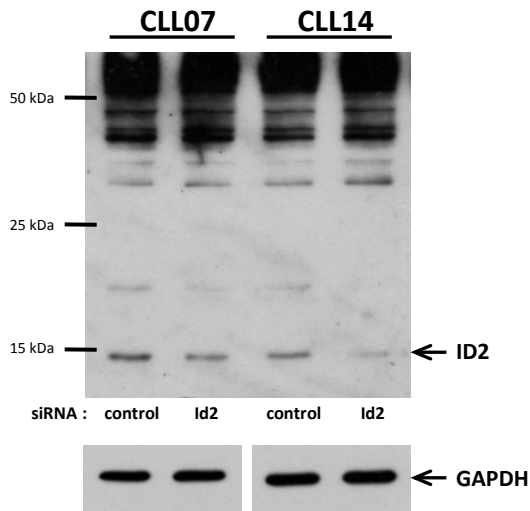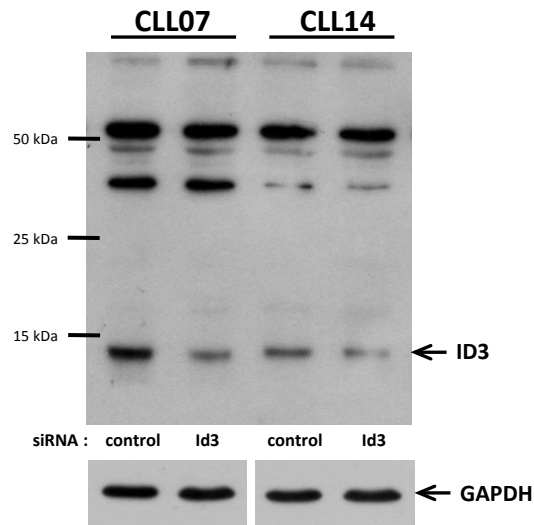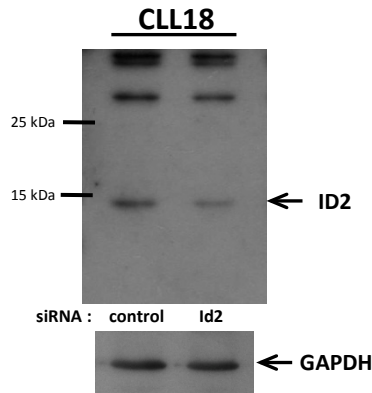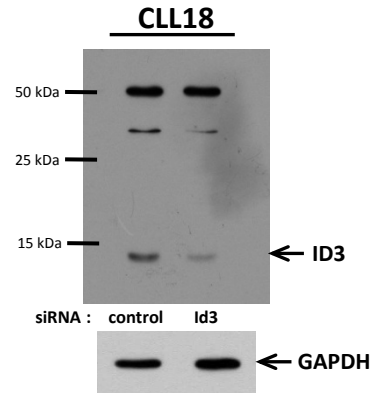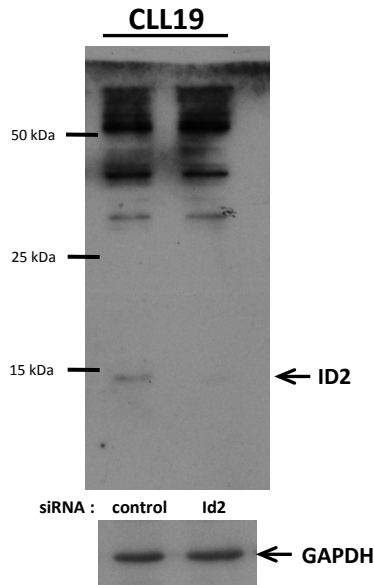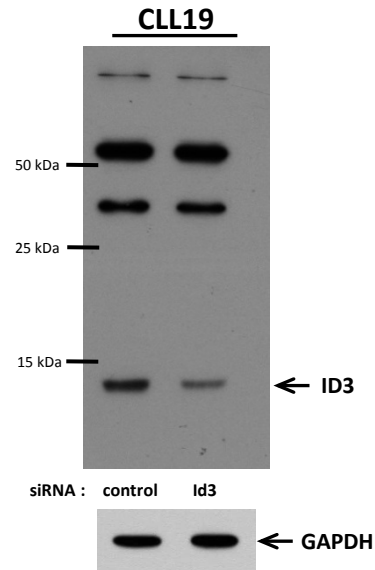

Supplement: Additional file 10: Figure S5. — Original scanned images of western blot analysis of ID protein expression in siRNA knock-down experiments. The images shown were used to compile Figure 10A&C in the main manuscript. The order of samples for the analysis of MEC1 cells is the same as in Figure 10. An indicative size marker scale in kDa is shown together with identities of the 36 kDa GAPDH and ID2/ID3 protein bands. The latter were verified in independent siRNA knock-down and transfection-over-expression experiments. Note that only the relevant sections of blots were re-probed with antibody for GAPDH. [file 12943_2014_286_MOESM10_ESM.pdf]

**A**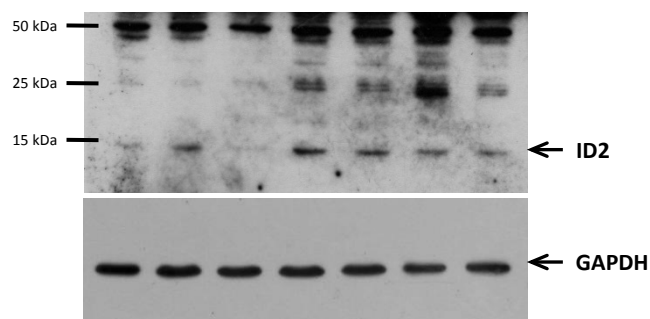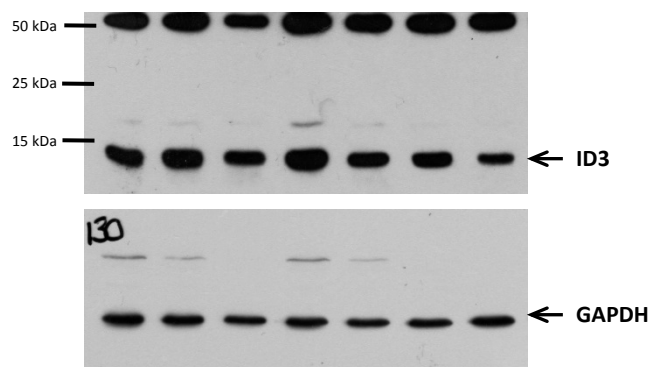**B**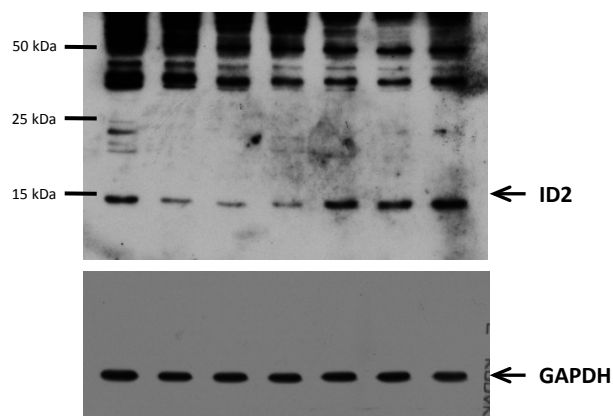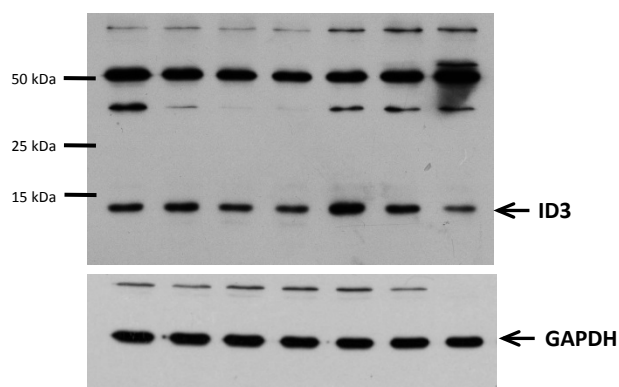**C**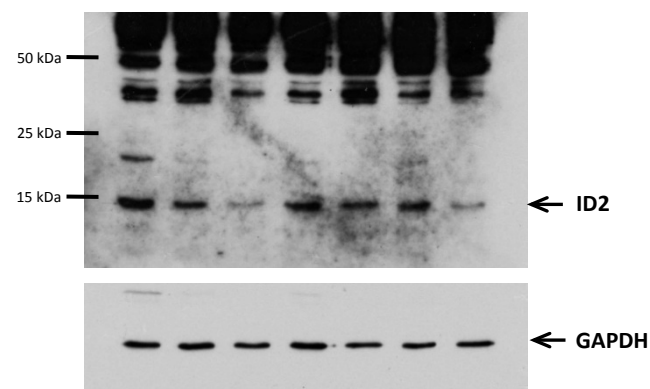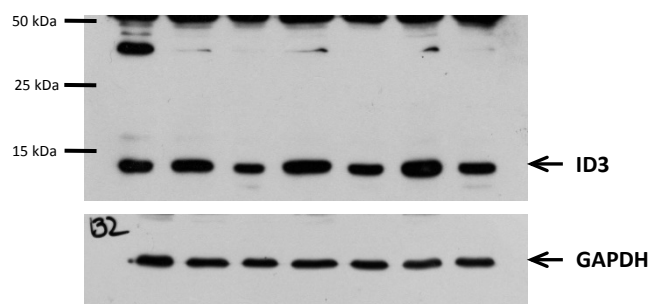**D**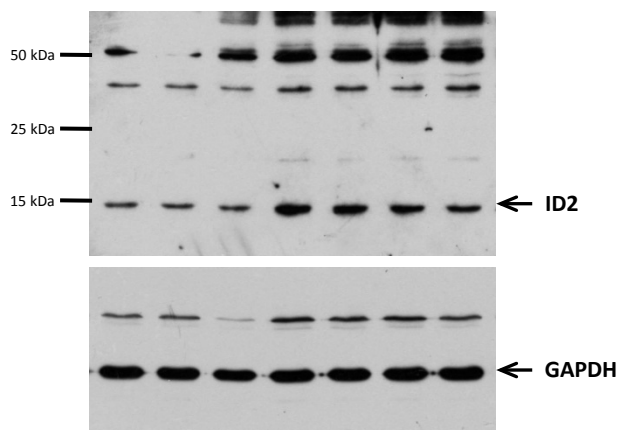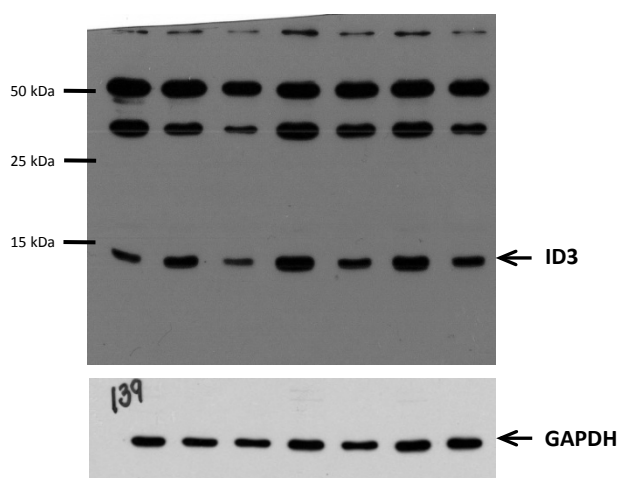

Supplement: Additional file 11: Figure S6. — Original scanned images of western blot analysis of ID protein expression in HUVEC co-culture experiments. The images shown in panels A-D were used to compile Figure 12 in the main manuscript. The order of CLL samples in each panel (A-D) corresponds to that in Figure 12. An indicative size marker scale in kDa is shown together with identities of the 36 kDa GAPDH and ID2/ID3 protein bands. The latter were verified in independent siRNA knock-down and transfection-over-expression experiments. Note that only the relevant sections of blots were re-probed with antibody for GAPDH. [file 12943_2014_286_MOESM11_ESM.pdf]

## CLL12 - PEITC

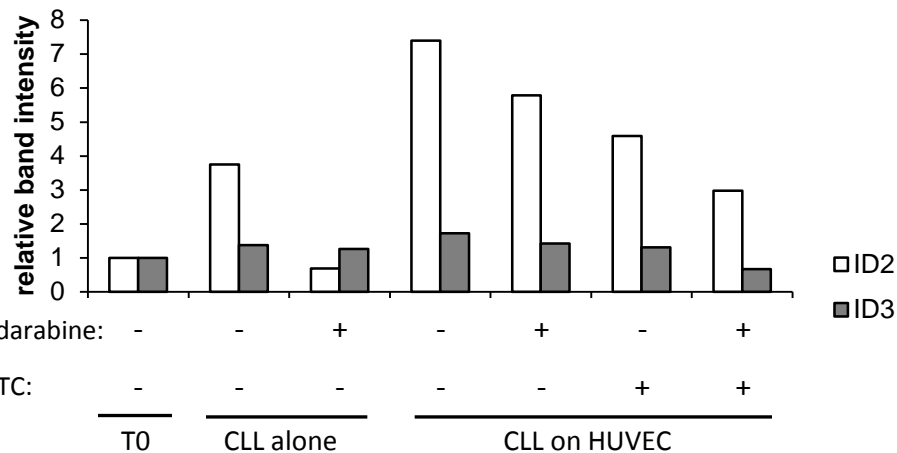

## CLL18 - PEITC

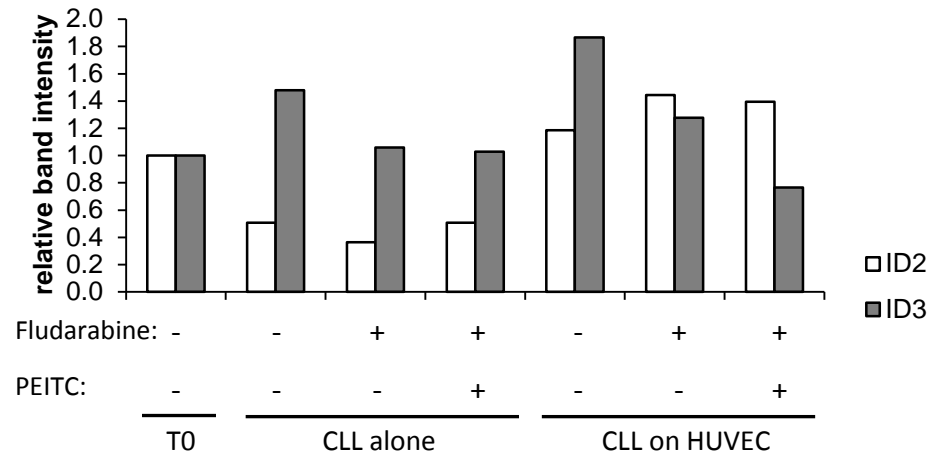

## CLL12 - GSH

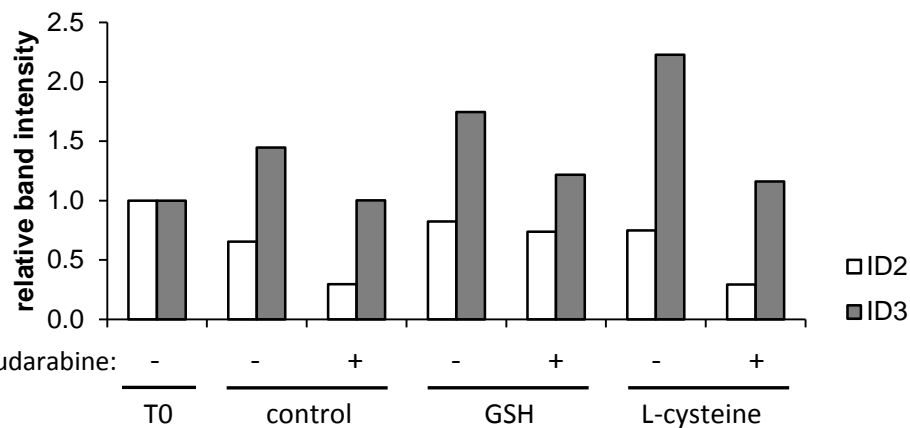

## CLL18 - GSH

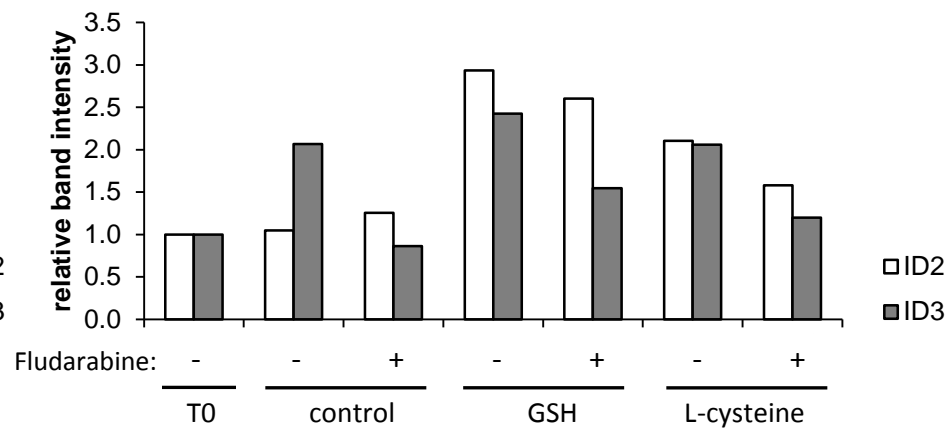

Supplement: Additional file 12: Figure S7. — Quantification of western blot analysis of ID protein expression levels in CLL cells following manipulation of intracellular GSH levels. Band intensities of the western data shown in Figure 12 of the main manuscript were quantified by densitometric scanning using ‘ImageJ’ software. Data were normalized to the GAPDH loading control and, for each ID protein, expressed as fold-change (relative band intensity) relative to the uncultured control (T0). [file 12943_2014_286_MOESM12_ESM.pdf]
