# Supplementary material for: ID helix-loop-helix proteins as determinants of cell survival in B-cell chronic lymphocytic leukemia cells in vitro
Source: Mol Cancer. 2015 Feb 3;14(1):30. doi: 10.1186/s12943-014-0286-9 (PMC4320821; doi:10.1186/s12943-014-0286-9)
Supplement: Additional file 7: Table S5. — Description of CLL patient characteristics. [file 12943_2014_286_MOESM7_ESM.doc]

**Table S5. Description of CLL patient characteristics.**

| **Patient number** | **WCC^1^** | **Age** | **Gender** | **Clinical stage** | **Treatment** | **Clinical response** |
| --- | --- | --- | --- | --- | --- | --- |
| **CLL06** | 243 | 74 | F | A | None | N/A^2^ |
| **CLL07** | 59 | 60 | M | A | None | N/A^2^ |
| **CLL08** | 91 | 53 | M | A | None | N/A^2^ |
| **CLL09** | 105 | 51 | M | A | None | N/A^2^ |
| **CLL10** | 47 | 70 | F | A | None | N/A^2^ |
| **CLL11** | 168 | 76 | F | A | None | N/A^2^ |
| **CLL12** | 132 | 80 | F | A | None | N/A^2^ |
| **CLL13** | 181 | 81 | F | Not determined | None | N/A^2^ |
| **CLL14** | 58 | 58 | M | A | None | N/A^2^ |
| **CLL15** | 86 | 75 | F | A | Chlorambucil | CR^3^ |
| **CLL16** | 72 | 61 | F | A | None | N/A^2^ |
| **CLL17** | 98 | 84 | F | A | Chlorambucil | Not declared |
| **CLL18** | 55 | 73 | M | A | Chlorambucil | PR^4^ |
| **CLL19** | 52 | 83 | M | Progressive | None | N/A^2^ |

^1^WCC, white cell count; ^2^N/A, not applicable; ^3^CR, complete response; ^4^PR, partial response.
